# Distributed control circuits across a brain-and-cord connectome

**DOI:** 10.1101/2025.07.31.667571

**Published:** 2025-08-02

**Authors:** Alexander Shakeel Bates, Jasper S. Phelps, Minsu Kim, Helen H. Yang, Arie Matsliah, Zaki Ajabi, Eric Perlman, Kevin M. Delgado, Mohammed Abdal Monium Osman, Christopher K. Salmon, Jay Gager, Benjamin Silverman, Sophia Renauld, Matthew F. Collie, Jingxuan Fan, Diego A. Pacheco, Yunzhi Zhao, Janki Patel, Wenyi Zhang, Laia Serratosa Capdevilla, Ruairí J.V. Roberts, Eva J. Munnelly, Nina Griggs, Helen Langley, Borja Moya-Llamas, Ryan T. Maloney, Szi-chieh Yu, Amy R. Sterling, Marissa Sorek, Krzysztof Kruk, Nikitas Serafetinidis, Serene Dhawan, Tomke Stürner, Finja Klemm, Paul Brooks, Ellen Lesser, Jessica M. Jones, Sara E. Pierce-Lundgren, Su-Yee Lee, Yichen Luo, Andrew P. Cook, Theresa H. McKim, Emily C. Kophs, Tjalda Falt, Alexa M. Negrón Morales, Austin Burke, James Hebditch, Kyle P. Willie, Ryan Willie, Sergiy Popovych, Nico Kemnitz, Dodam Ih, Kisuk Lee, Ran Lu, Akhilesh Halageri, J. Alexander Bae, Ben Jourdan, Gregory Schwartzman, Damian D. Demarest, Emily Behnke, Doug Bland, Anne Kristiansen, Jaime Skelton, Tom Stocks, Dustin Garner, Farzaan Salman, Kevin C. Daly, Anthony Hernandez, Sandeep Kumar, The BANC-FlyWire Consortium, Sven Dorkenwald, Forrest Collman, Marie P. Suver, Lisa M. Fenk, Michael J. Pankratz, Gregory S.X.E. Jefferis, Katharina Eichler, Andrew M. Seeds, Stefanie Hampel, Sweta Agrawal, Meet Zandawala, Thomas Macrina, Diane-Yayra Adjavon, Jan Funke, John C. Tuthill, Anthony Azevedo, H. Sebastian Seung, Benjamin L. de Bivort, Mala Murthy, Jan Drugowitsch, Rachel I. Wilson, Wei-Chung Allen Lee

**Affiliations:** 1 Department of Neurobiology, Harvard Medical School,Boston, MA, USA; 2 Centre for Neural Circuit and Behaviour, University of Oxford, Oxford, UK; 3 Present address: Neuroengineering Laboratory, BrainMind Institute and Institute of Bioengineering, EPFL, Lausanne, Switzerland; 4 Present address: Department of Molecular and CellularBiology, Harvard University, Cambridge, MA, USA; 5 Present address: Center for Brain Science, HarvardUniversity, Cambridge, MA, USA; 6 Princeton Neuroscience Institute, Princeton University,Princeton, NJ, USA; 7 Yikes LLC, Baltimore, MD, USA; 8 Aelysia LTD, Bristol, UK; 9 Department of Organismic and Evolutionary Biology,Harvard University, Cambridge, MA, USA; 10 Center for Brain Science, Harvard University, Cambridge,MA, USA; 11 Present address: Psychology Department, ColoradoCollege, Colorado Springs, CO, USA; 12 Eyewire, Boston, MA, USA; 13 Neurobiology Division, MRC Laboratory of MolecularBiology, Cambridge, UK; 14 Genetics Department, Leipzig University, Leipzig,Germany; 15 Zoology Department, University of Cambridge, Cambridge,UK; 16 Department of Molecular Genetics and Cell Biology,The University of Chicago, Chicago, IL, USA; 17 Department of Neurobiology and Biophysics, Universityof Washington, Seattle, WA, USA; 18 Department of Biology, West Virginia University,Morgantown, WV, USA; 19 Department of Biology, University of Nevada Reno,Reno, NV, USA; 20 Department of Biological Sciences, Vanderbilt University,Nashville, TN, USA; 21 Max Planck Institute for Biological Intelligence,Martinsried, Germany; 22 Institute of Neurobiology, University of Puerto Rico Medical Sciences Campus, San Juan, Puerto Rico; 23 Zetta AI LLC, Sherrill, NY, USA; 24 School of Informatics, University of Edinburgh,Edinburgh, UK; 25 Japan Advanced Institute of Science and Technology(JAIST), Nomi, Japan; 26 Molecular Brain Physiology and Behavior, LIMES Institute,University of Bonn, Bonn, Germany; 27 Present address: Department of Neurobiology, HarvardMedical School, Boston, MA, USA; 28 Department of Neuroscience, West Virginia University,Morgantown, WV, USA; 29 Allen Institute for Brain Science, Seattle, WA,USA; 30 School of Neuroscience, Virginia Tech, Blacksburg,VA, USA; 31 Department of Biochemistry and Molecular Biology, University of Nevada Reno, Reno, NV, USA; 32 Neurobiology and Genetics, Theodor-Boveri-Institute,Biocenter, Julius-Maximilians-University of Würzburg, Am Hubland, Würzburg, Germany.; 33 HHMI Janelia, Ashburn, VA, USA; 34 Computer Science Department, Princeton University,Princeton, NJ, USA; 35 F.M. Kirby Neurobiology Center, Boston Children'sHospital, Harvard Medical School, Boston, MA, USA

## Abstract

Just as genomes revolutionized molecular genetics, connectomes (maps of neurons and synapses) are transforming neuroscience. To date, the only species with complete connectomes are worms^1–3^ and sea squirts^4^ (10^3^-10^4^ synapses). By contrast, the fruit fly is more complex (10^8^ synaptic connections), with a brain that supports learning and spatial memory^5,6^ and an intricate ventral nerve cord analogous to the vertebrate spinal cord^7–11^. Here we report the first adult fly connectome that unites the brain and ventral nerve cord, and we leverage this resource to investigate principles of neural control. We show that effector cells (motor neurons, endocrine cells and efferent neurons targeting the viscera) are primarily influenced by local sensory cells in the same body part, forming local feedback loops. These local loops are linked by long-range circuits involving ascending and descending neurons organized into behavior-centric modules. Single ascending and descending neurons are often positioned to influence the voluntary movements of multiple body parts, together with endocrine cells or visceral organs that support those movements. Brain regions involved in learning and navigation supervise these circuits. These results reveal an architecture that is distributed, parallelized and embodied (tightly connected to effectors), reminiscent of distributed control architectures in engineered systems^12,13^.

A coherent understanding of the embodied nervous system remains a central challenge of neurobiology. The fruit fly *Drosophila melanogaster* is the most complex organism for which this milestone is currently within reach. Recent work has yielded connectomes for the adult *Drosophila* brain^14–17^ and ventral nerve cord (VNC)^7–11^. These structures are analogous to the brain and spinal cord of vertebrates, but they contain fewer neurons, making them tractable for complete connectomes (brain: ~140,000 neurons, VNC: ~20,000 neurons). The fly brain and VNC are connected by ~1300 descending neurons (DNs)^18–21^ and ~1900 ascending neurons (ANs)^21–25^. However, the existing fly brain^14–17^ and VNC^7–11^ connectomes were collected separately, and so DNs and ANs are fragmentary in these datasets, though cross-mapping of some cell types have allowed some ‘bridging’ analyses^21^. A unified *Drosophila* connectome would allow us to trace the pathways that connect the brain, VNC and body.

Such a connectome would also shed light on the architecture of behavioral control. Different regions of the central nervous system (CNS) have specialized functions—and this is true in arthropods just as in vertebrates^26^—but we lack a detailed understanding of the overall control architecture in any complex neural system. In principle, behavioral control might flow through a central pathway for perception, action selection and motor coordination; alternatively, it might be decentralized and distributed across many feedback control modules that are loosely coupled in a hierarchical manner. These alternative scenarios are debated in the literature on vertebrate intelligence, insect intelligence and artificial intelligence^13,27–29^. A unified adult *Drosophila* connectome would place important constraints on this debate. Adult flies are also limbed organisms that solve many of the basic control problems that confront other limbed species, including vertebrates^30^.

In this study, we describe the first unified and embodied brain-and-cord connectome of an adult fly. To analyze this dataset, we develop an influence metric to predict the functional connection between any pair of cells, and we apply this at scale to the entire nervous system. We show that the strongest influences on effectors (motor neurons, endocrine cells and efferent neurons targeting the viscera) are generally local sensory signals, forming a distributed set of tight feedback loops. Long-range connections involving ANs and DNs coordinate these local loops. Many of these AN/DN circuits can be linked to specific behaviors, such as escape, feeding, reproduction and locomotion. We describe the interactions between these circuits, and we explicitly link these circuits to supervisory brain regions involved in learning and navigation. Our results establish clear empirical support for theories of behavioral control organized around distributed sensory-motor modules, where “cognitive” regions are supervisory but not essential for action.

## Results

### An open-source brain-and-nerve-cord connectome

**Extended Data Fig. 1:**
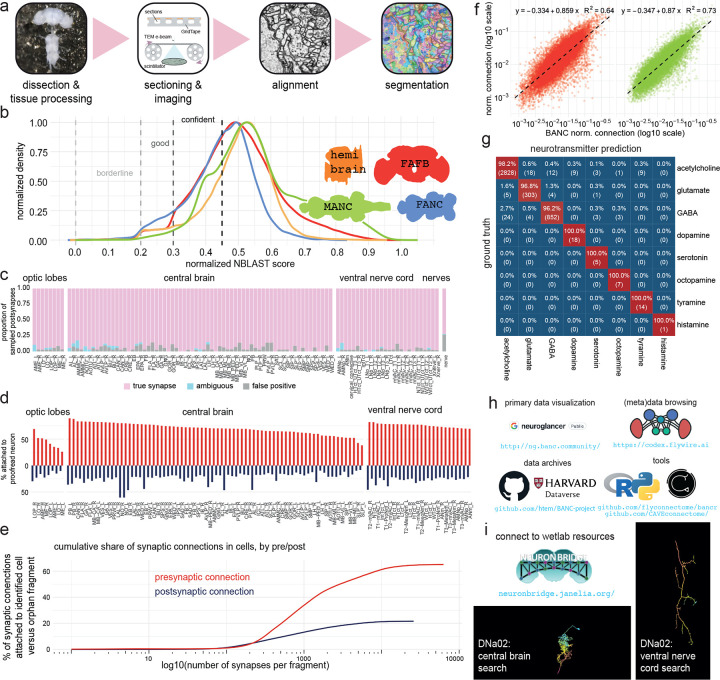
central nervous system connectome generation, quality, and neuron identification a.Workflow for serial EM dataset generation. The specimen is dissected and prepared for sectioning and EM imaging. Acquired EM micrographs are then aligned into a dataset, which is subsequently segmented into cellular fragments. b.Density of the normalized NBLAST scores^39^ of ‘proofread’ neurons^15^ in the BANC against all neurons in other connectomic datasets (different colors). We consider normalized NBLAST scores > 0.3 as high and suggest score bins to help guide data users (dashed lines). Normalized NBLAST scores are “raw” NBLAST scores divided by self-match score. All density curves are normalized to their own peak. c. We sampled 4648 postsynaptic links evenly across 67 standard neuropils^35,36^ for a false positive review (_L, left neuropil, _R, right neuropil). d.Attachment rates for presynaptic (red) and postsynaptic (navy) links to an identified cell (neuron, glia) across neuropils. We used the BANC synapse version: synapses_250226. e.The cumulative share of pre- and postsynaptic links in identified cells versus orphan fragments (not part of an identified cell). Plot is by fragment size as inferred by number of links on fragment (version 626). f. Scatter plots show the correlation between matched pairs of connected cell types in the BANC versus FAFB^15^ and MANC^11^ (and the most complete extant connectomes). Each point is a cell-type-to-cell-type normalized connection (synaptic connections from source-to-target / total number of postsynaptic links on the target cell type). FAFB-BANC: 34174 matched cell type connections, MANC-BANC: 29350 matched cell type connections. g.Confusion matrix of neurotransmitter prediction evaluated at the level of whole neurons on the held-out test set. Whole neuron prediction is based on the summed classification probabilities across all presynaptic links, selecting the most confident class. The ground-truth included 20572 neurons (from 2900 cell types, see Methods), of which 16448 were used for training and 4124 for testing. h.Users can browse BANC data via Codex (codex.flywire.ai/banc), and they can download data for programmatic analysis (via Codex^15^, CAVE^33^, and Dataverse at https://doi.org/10.7910/DVN/8TFGGB). i. Color-depth MIPs^281^ (maximum intensity projection images where color encodes depth) in JRC2018U space^261^ for BANC dataset neurons (version 626) available from our Dataverse archive. These can be used to search for genetic driver lines enabling functional investigation into BANC neurons, for example using NeuronBridge^262^. Examples are shown for a specific cell type (DNa02).

**Figure 1:**
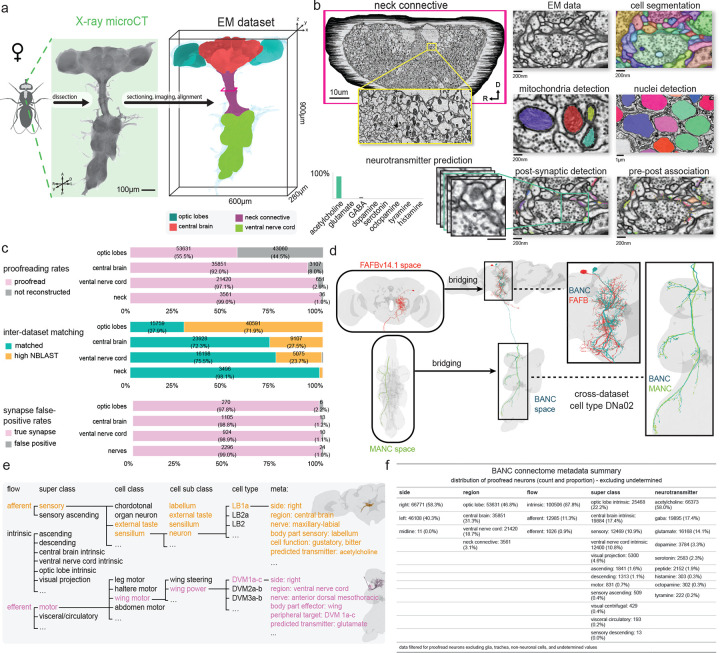
An open-source brain-and-nerve-cord connectome. a. (*left*) X-ray micro-computed tomography (microCT) projection of the BANC sample following dissection, staining, and embedding for EM. (*right*) Surface mesh rendering of the CNS EM dataset with regions colored. A: anterior, P: posterior, D: dorsal, V: ventral, L: left, R: right. b. (*top left*) Aligned EM micrographs through a cross-section of the neck connective (y=92500) (magenta box in (a)). D: dorsal, R: right. (*yellow box*) Zoom-in of the EM data. (*columns to right*) Example EM image data from the BANC dataset. Neurons were automatically segmented using convolutional neural networks (CNNs)^31,76^, with each segmented cell shaded with a different color. Mitochondria (x: 137533, y: 35220, z: 2493) and nuclei (x: 192977, y: 51679, z: 2493) (both overlaid with different colors) were segmented^77^. Postsynaptic locations (shaded with different colors, example: x: 140988, y :36705, z: 2498) were automatically predicted and presynaptic locations (end of yellow lines) were automatically assigned using CNNs^45^. (*bottom left*). The predicted neurotransmitter for the selected synapse (center of the green box) is acetylcholine. c. (*top*) Fraction of proofread neurons in gross divisions of the CNS. Neurons are labeled as proofread when their primary neurites or ‘backbones’ have been reviewed^15^. (*middle*) Fraction of proofread neurons in the BANC matched with neurons in other connectomes, by gross divisions of the CNS. Morphological cell type level matches were confirmed by experts (teal), or matched to a likely class based on high NBLAST scores^39^. (*bottom*) Fraction of true and false positive synapse predictions in different divisions of the CNS. Full CNS inventory inferred from summing counts from FAFB and MANC, and subtracting photoreceptors not captured by BANC (11468). d. Neurons were matched to metadata from previous projects by transforming their morphologies from other connectomes^8,10,11,14,15,17^ into BANC space^78^. We used NBLAST^39^ to identify potential morphological matches. An example with DNa02 is shown, illustrating the process. Neuroglancer link for morphology, Codex link for metadata/connectivity. e. Hierarchy of cell annotations, based on previous work^17,79^, but adopting clearer terms. Exemplified for LB1a (Neuroglancer link, Codex search) and DVm1a-c (Neuroglancer link, Codex search). See (Supplementary Data 1). f. The proportion of proofread neurons (of 114518) in the BANC by metadata label. Fast-acting neurotransmitter identities are assigned by our native BANC neurotransmitter predictions, based on^46^. The ‘peptide’ class was added in cases where evidence from the literature supports neuropeptide expression, but our prediction is for a monoamine. In these cases we suspect the predictions are more likely to be incorrect^46^. It is not meant to represent the number of peptidergic neurons, which would be far larger..

We generated a serial-section electron microscopy (EM) volume comprising the CNS of an adult female *D. melanogaster* at synapse resolution (4×4×45 nm^3^) (Fig. 1a). Using our semi-automated sectioning and imaging platform (GridTape^7^) (Extended Data Fig. 1a), we collected 7,010 serial sections onto film-coated tape, compatible with transmission EM. This approach enabled visualization of fine neural processes (<200 nm), synaptic vesicles (~40 nm) and synaptic clefts (~10 nm). After imaging each section, we computationally reassembled the entire Brain and Nerve Cord dataset (BANC, pronounced “bank”) into a 3D volume^8,31^. We then used convolutional neural networks (CNNs) to automatically segment and reconstruct individual cells^8,31^, nuclei, and mitochondria (Fig. 1b). To proofread and annotate the expected ~160,000 neurons^10,15^ in the dataset, we followed the approach created by FlyWire for the whole-brain connectome (FAFB-FlyWire)^15,32,33^. We used automatically identified nuclei to account for all neurons with their cell bodies in the CNS. For neurons with cell bodies outside the CNS (e.g., sensory neurons), we manually identified 48 nerves^34–37^ and verified that each axon in these nerves was associated with a segmented neuron. For neurons traversing the neck connective, we verified that every axon at both anterior and posterior neck levels was associated with a segmented neuron. A team of 155 proofreaders corrected errors in the automatic segmentation over about 2 years, a total effort of ~30 work-years (Fig. 1c).

We assigned cell type labels by automatically identifying potential matches between BANC neurons and earlier datasets^8–10,14,15,17,38^, based on neuron morphology and position (using NBLAST^39^, Fig. 1c–e, Extended Data Fig. 1b) and based on connectivity^40^ (A.M., C.K.S., et. al., in preparation). We then manually reviewed and corrected these cell type matches; this process is largely complete but is still ongoing in the left optic lobe (Fig. 1c). Some neurons are still not cross-matched (26% of BANC neurons excluding the optic lobes), and some of these neurons likely cannot be matched even with more effort, due to inter-individual variability in cell morphology^41–43^. Inter-individual variability can result from genetic variation, developmental noise and limitations in data quality or reconstruction. Importantly, in the course of making cell type assignments, we generated the first comprehensive accounting of DN and AN cell types, and we matched AN/DN cell-type labels across the existing whole-brain connectome^15^ and VNC connectomes^8–10^.

To automatically identify synapses in the BANC, we trained another CNN^44,45^ to predict pre- and postsynaptic locations with high accuracy (F-score: .79, precision: .68, recall: .95; Fig. 1b,c, Extended Data Fig. 1c). Overall, 65% of detected presynaptic links are attached to identified cells (Extended Data Fig. 1d,e). Comparing the normalized synaptic count between all pairs of cross-matched, identified cell types in the CNS revealed strong concordance between the BANC and other adult fly connectomes (Extended Data Fig. 1f).

We used another CNN to predict the neurotransmitter released by each neuron^46^. Our identifications of neurons releasing acetylcholine, glutamate, GABA, dopamine, serotonin and octopamine largely agree with previous predictions^46^. We also used this approach to identify cells that release tyramine and histamine, which have not been previously incorporated into automatic neurotransmitter predictions (Extended Data Fig. 1g).

Next, we identified many cell types linking the CNS with the rest of the body (Fig. 1c–f). To do so, we annotated BANC cells based on literature review, neuron matching and refined labels from prior connectomes. For example, we identified motor neurons targeting muscles of the limbs, antennae, eyes, neck, crop, pharynx, proboscis, salivary glands and uterus^8,10,47–54^. We found putative sensory nociceptors from the abdomen^10^ (J.J. & J.C.T., in preparation), sensory neurons from the aorta^55^, the cibarium^56^ (the pre-oral food chamber), putative oxygen-sensing neurons in the abdomen^57,58^ and sensory neurons from the abdominal terminalia^52,59^. We identified multiple distinct types of endocrine cells in the brain and VNC, many of which could be matched with the neuropeptides they release^58,60–64^ and their sites of action, including the ureter^65,66^, neurohemal release sites, the digestive tract^55,67^, and the reproductive tract^68–70^. We also identified chemosensory, tactile and proprioceptive afferents from the head, eyes, antennae, proboscis, legs, abdomen, wings and halteres^10,37,71–75^. Taken together, these cell type identifications make the BANC a highly ‘embodied’ connectome, with explicit connections to specific muscles, sense organs and viscera throughout the body.

Our ability to describe all these connections relied crucially on BANC being an open science effort^32^ since July 2023, and this project continues to grow with ongoing community input. Users can visualize the latest version of our data via Neuroglancer^80^ and add annotations through CAVE^33^. Users can also browse metadata and connectivity data via FlyWire Codex^15^ (codex.flywire.ai/banc) and CAVE^33^, as well as programmatically^78^ and via direct downloads^81^ (Extended Data Fig. 1h,i). We have also modified typology annotations for the whole-brain (FAFB) and VNC (MANC) connectomes^10,11,15–17^ to facilitate comparisons between these datasets and our work in BANC (Supplementary Data 1–3).

### A metric of influence

To interpret a whole-CNS connectome, we need a way to estimate the influence of cell A on cell B, for any pair of cells. To date, there has been no computationally efficient method of estimating these influences. Efficiency is crucial, as there are billions of pairwise interactions between cells in the full CNS. It would be ideal to precompute all these influences, so that users can simply query any cell pair of interest.

**Extended Data Fig. 2:**
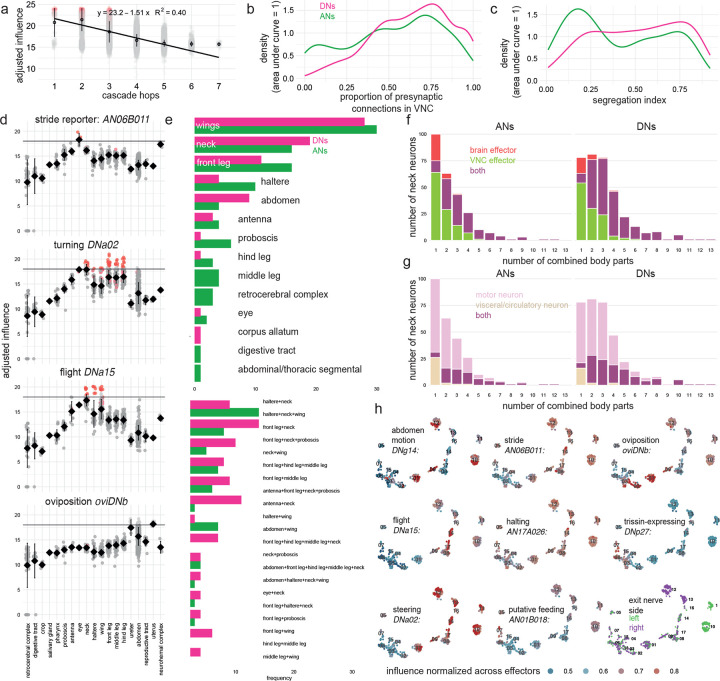
Individual DNs and ANs often influence effectors in multiple body parts. a. Fig. 2b shows that the adjusted influence is proportional to ‘layers’ of a published graph traversal model^41^ applied to the FAFB dataset^17^. Here we show that the adjusted influence is also proportional to the output of a different published layering algorithm^22^. As in Fig. 2b, we used olfactory seeds annotated in the FAFB dataset. b. Distribution of presynaptic links in the VNC versus the brain, for all DNs (1313 cells) and ANs (1841 cells) in the BANC dataset. c. Distribution of segregation index^282^ values for these same DNs and ANs. Segregation index is a measure of polarization which quantifies the entropy of pre- and postsynaptic connections between the axonal and dendritic compartments of a neuron. A segregation index closer to 1 indicates a more polarized neuron. d. Here we chose three DNs and one AN that have clear behavioral effects, and we examined their adjusted influence on effector cells in different body parts. Within each subplot, each point is an effector cell, with direct connections in red. The horizontal line marks a value of 17.18, which we take as a conservative cutoff for “high influence” (see note below). All four cells have some effector influence above this cutoff. For each cell, the above-cutoff effector influences are compatible with the cell’s function. e. After discarding connections below this cutoff, we counted the number of AN and DN cell types that influence effectors in single body parts (top) or multiple body parts (bottom). The bottom plot shows only the most common 20 combinations of body parts. f. The number of AN and DN cell types that combine different numbers of body parts. Gross CNS division for combined effectors shown in color (‘both’ can appear when only one body part is targeted, because neck motor neurons can exist in both the brain and VNC^53^). g. Same as (f), but color indicates combinations across motor classes and visceral/circulatory classes. h. The effector cell map from Fig. 2i, color-coded by adjusted influence from example ANs and DNs. Bottom right, cells are color-coded by the side of the CNS on which their efferent axon exits. Note, we chose this adjusted influence cutoff because it is the “elbow” in the cumulative distribution of AN/DN-to-effector adjusted influences involving DNs and ANs with known behavioral functions; DNs and ANs used to identify this elbow were DNa02^104^, DNa01^104^, DNp01^115^, DNp02^116^, MDN (DNp50)^96^, DNp42^103^, DNg97^97^, DNg100^97^, DNg12^101^, DNg62^98^, DNp07^100^, DNp10^100^, DNg14^95^, DNa15^114^, DNb01^114^, DNp37^133^, oviDNb^70^, DNp20^107^, DNp22^107^, DNp25^283^, DNp44^283^, DNp27^225^, AN17A026^108^ and AN19A018^97^.

**Figure 2:**
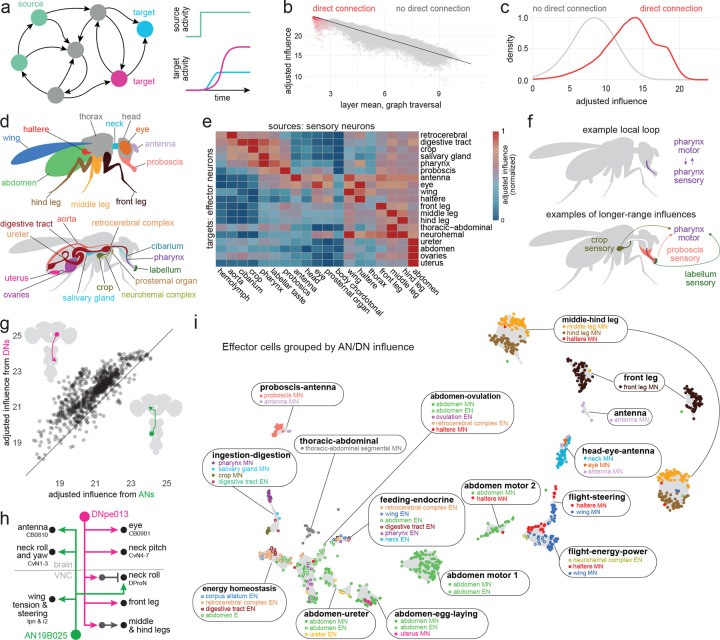
Linking sensors and effectors through local and long-range circuits. a. The influence of source cells on target cells is estimated via linear dynamical modeling. b. Adjusted influence (see Methods) is proportional to the number of network ‘layers’ in a graph traversal model^41^. Direct and indirect connections are shown in red and gray, respectively. Here the source neurons are olfactory receptor neurons in the FAFB dataset, following previous work^17^, and adjusted influence is averaged over the number of neurons in the source and target groups. Regression line in black (R^2^=0.94, n = 94278). c. Distribution of adjusted influence scores between all ANs (1841) and DNs (1313) and all other neurons (155936) in the dataset. Direct and indirect connections are shown in separate histograms, with the peak of each histogram normalized to its own maximum. d. Schematic of body parts associated with annotated effector cells in the BANC. Not all neurohemal organs shown. Neuroglancer link, explore on Codex here. e. Mean adjusted influence of sensory cells (columns) on effector cells. Sensory and effector cells are pooled by body part. Each row is minmax normalized to the same range (0–1). This plot summarizes data from 14410 sensory cells and 1026 effector cells. We omitted 3188 putative sensory cells whose corresponding organs could not be identified. f. Schematic: an example local loop (top) that is also linked to specific sensors via long-range connections (bottom). g. Scatterplot showing the mean adjusted influence on each effector cell from DNs versus ANs. Black, unity line. Insets: a DN soma is located in the head, whereas an AN soma is located in the body. h. An example AN and DN with strong adjusted influence on effector cells in multiple body parts. Neuroglancer link, Codex network. i. UMAP embedding of effector cells, based on the cosine similarity between the adjusted influences these cells receive from individual ANs and DNs. The major cell types in each effector cell group are listed (MNs, 833 motor neurons; ENs, 193 endocrine neurons some of which are putative). Neuroglancer link, Codex search. See (Supplementary Data 5).

To tackle this problem, we developed an approach based on linear dynamical modeling^82–85^. Specifically, to compute the influence of one or more source neurons on any target neuron(s), we simulate the effect of injecting a sustained step of activity into the source neurons, taking every downstream neuron’s activity as the weighted sum of its inputs. The weight is the number of synapses in that input connection^86^, as a fraction of the postsynaptic cell’s total synaptic input. For a target cell of interest, we take its steady-state activity (Fig. 2a), log-transform it, and add a constant to ensure that the result is nonnegative. The metric (called ‘adjusted influence’) is approximately linear with network distance from source to target (Fig. 2b, Extended Data Fig. 2a). Indeed, adjusted influence is in excellent agreement with previous network distance metrics^15,22,41^. Like previous distance metrics^15,22,41^, adjusted influence is an unsigned quantity. However, unlike those metrics, our metric is deterministic, linear and scalable. This allowed us to precompute the pairwise adjusted influence of all individual neurons in the entire CNS onto all other individual neurons, yielding 24 billion scores in total. Across the CNS, the modal adjusted influence score is 14 for direct connections and 8 for indirect connections (Fig. 2c). All scores are available to users via codex.flywire.ai/banc.

In the following sections, we say A “influences” B, as shorthand for a high adjusted influence score (A→B). These scores do not demonstrate functional connections, and they are no substitute for experiments. The value of these scores is that they allow us to make provisional inferences on a large scale. In the sections that follow, we will use influence scores to make inferences, and to bolster these inferences, we will show example circuit motifs. These inferences are merely predictions, and their value is to generate testable hypotheses.

### Modules for local feedback control

Other large-scale connectome analyses have focused mainly on cells deep in the CNS^5,6,87^. Here, we take a complementary approach; we start by focusing on sensors and effectors. A ‘sensor’ is a presumptive peripheral sensory neuron (either external or internal) and an ’effector’ is a presumptive motor neuron, endocrine cell, or an efferent neuron targeting the viscera (Fig. 2d). Importantly, sensors are distributed across the body, and effectors are widely distributed as well: the brain contains motor neurons that control the eyes, antennae, mouth parts, as well as the foregut^88^, while the VNC contains motor neurons that control the limbs, abdomen, reproductive organs, and hindgut^7^. Similarly, endocrine cells are found in both the brain and the VNC^89,90^. As an embodied brain-and-VNC connectome, the BANC offers a new opportunity to reconsider sensor-effector relationships.

As a general rule, we found that effector cells receive their strongest influence from sensors in the same body part (Fig. 2e). To take an arbitrary example, we find that pharynx motor neurons are most strongly influenced by pharynx sensory cells. Ongoing pharynx movements will also immediately alter the activity of pharynx sensory neurons, and so these motor neurons form a tight reciprocal feedback loop with local sensory neurons (Fig. 2f). Local feedback is useful because it minimizes delays^71,91,92^. Previous work has described local feedback loops in proboscis control^47^, enteric control^55^, and VNC premotor networks^9,93^, and our analysis extends this work to argue that tight local feedback is a systematic principle across the CNS.

At the same time, the BANC dataset also shows that each local loop is influenced by a select group of more distant sensors (Fig. 2e). For example, pharynx motor neurons are influenced by sensors in the crop, labellum, and proboscis. These longer-range connections can also be seen as forming feedback loops: for example, pharynx movements during feeding should trigger not only immediate sensory signals in the pharynx, but also more delayed sensory signals in the crop, which might then (for example) limit feeding if the crop is filling too quickly (Fig. 2f). In this way, long-range loops can provide important feedback signals that local loops cannot directly access^91^. The BANC dataset shows that long-range influences are generally weaker than local influences (Fig. 2e), which implies that local loops are the core elements of behavioral control, with a secondary role for long-range loops.

### Linking DNs and ANs to effectors

Thus far, we have seen evidence for strong local feedback loops. These local loops are linked by selective longer-range sensory-motor connections. To better understand these long-range connections, we focused on the neurons that link the brain with the VNC, namely DNs and ANs.

It is sometimes suggested that DNs send motor commands from the brain to the VNC, whereas ANs send sensory signals and predictive motor signals back from the VNC to the brain^24,94^. But recent work has shown that ANs can also form output synapses in the VNC^9,10^, while DNs can form output synapses in the brain^21,95^. The BANC dataset allows us to reconstruct DNs and ANs comprehensively, and it shows clearly that both DNs and ANs have substantial output in both the brain and the VNC (Extended Data Fig. 2b,c). Moreover, the BANC dataset shows that most effector cells are influenced by both DNs and ANs (Fig. 2g). The majority of individual DNs exert influence over effector cells in multiple body parts, and the same is true of ANs (Extended Data Fig. 2d–g). For example, DNpe013 influences motor neurons in the eyes, neck and legs, whereas AN19B025 influences motor neurons controlling the eyes, antennae, neck and wings (Fig. 2h). Together, all these observations imply that DNs and ANs work together to coordinate motor patterns and internal organs in different body parts.

Next, we asked how DNs and ANs organize effector cells. We computed the influence of every DN and AN on every effector cell, and we divided effector cells into groups according to the influence they receive. This effector cell map (Fig. 2i, Extended Data Fig. 2h) identifies sets of coordinated motor neurons and endocrine neurons. For example, this map shows DNs and ANs co-regulate pharynx motor neurons, salivary gland motor neurons, crop motor neurons, and endocrine cells of the digestive tract; we call this the “ingestion-digestion group”. Similarly, DNs and ANs coordinate wing power motor neurons, haltere motor neurons and endocrine cells of the neurohemal complex; we call this the “flight-energy-power group”. As these examples illustrate, DNs and ANs often unite cells in different body parts (Fig. 2i).

To recap, we find that many individual DNs and ANs have distributed patterns of influence over effector cells. Both DNs and ANs are positioned to coordinate the actions of effectors across the body. Finally, DNs and ANs coordinate distinct groups of endocrine cells and motor neurons, allowing the body’s internal state to be coordinated with specific motor patterns.

### Clustering DNs and ANs into behavior-centric modules

**Extended Data Fig. 3:**
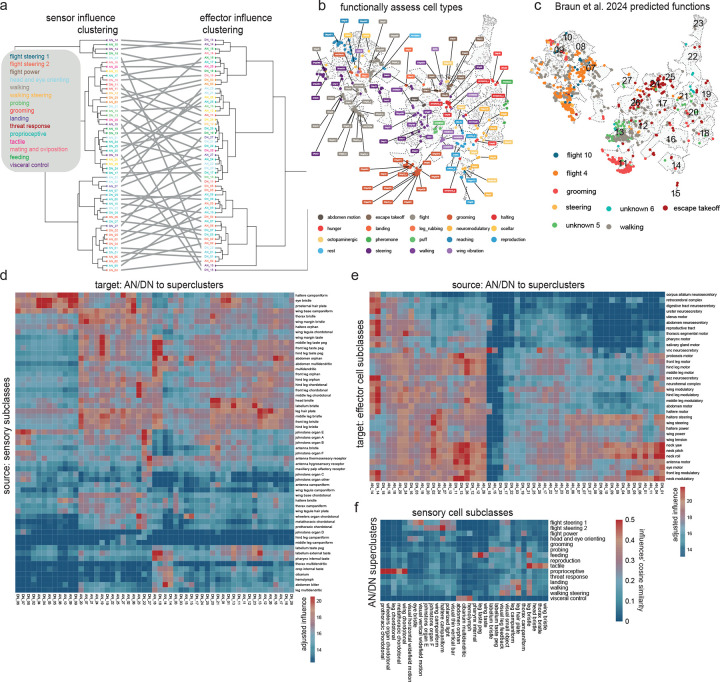
Influence streams to and from AN/DN clusters a. Tanglegram showing the relationship between two methods of sorting AN/DN clusters (Fig. 3a). The left dendrogram sorts clusters based on the similarity of their adjusted influences from sensor cell subclasses. The right dendrogram sorts clusters based on the similarity of their adjusted influence to effector cell subclasses (right). Colors denote superclusters. b. Names of studied cell types in the field, and their positions in our UMAP space, built by AN/DN direct connectivity to other neurons of the CNS. c. Our AN/DN map from Fig. 3a, with functions assigned by Braun et al. (2024)^95^. This earlier work only used direct FAFB DN-DN connectivity, and as a result, functional information was more limited than it is now. d. Adjusted influence from sensory neuron subclasses onto AN/DN neuron clusters. e. Adjusted influence from AN/DN clusters onto effector cell subclasses. f. Similarity of adjusted influence between specific sensory neurons and superclusters. Superclusters are rows; sensory neurons are columns.

**Extended Data Fig. 4:**
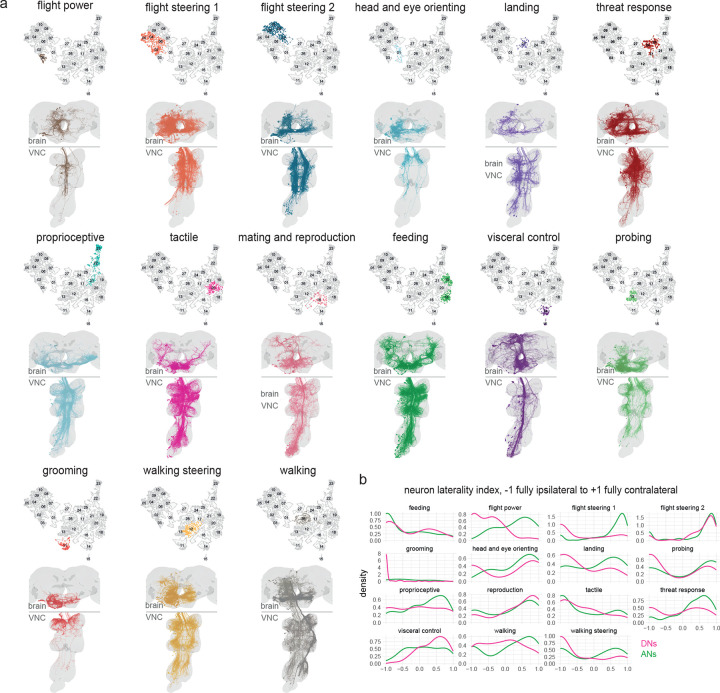
AN/DN morphologies by supercluster a. Each subpanel shows all right-side neurons from one AN/DN supercluster in the UMAP embedding. Neuroglancer links for flight power, flight steering 1, flight steering 2, head and eye orienting, landing, threat response, proprioceptive, tactile, mating and reproduction, feeding, visceral control, probing, grooming, walking steering and walking. b. Distribution laterality index values, for each AN/DN supercluster. Each synaptic connection is signed by the anatomical side of BANC in which it is found (−1 for left, +1 for right). Laterality index is: 1 - abs(mean of the postsynaptic score - mean of the presynaptic score). Each distribution is scaled so that the area under the curve is 1.

**Figure 3:**
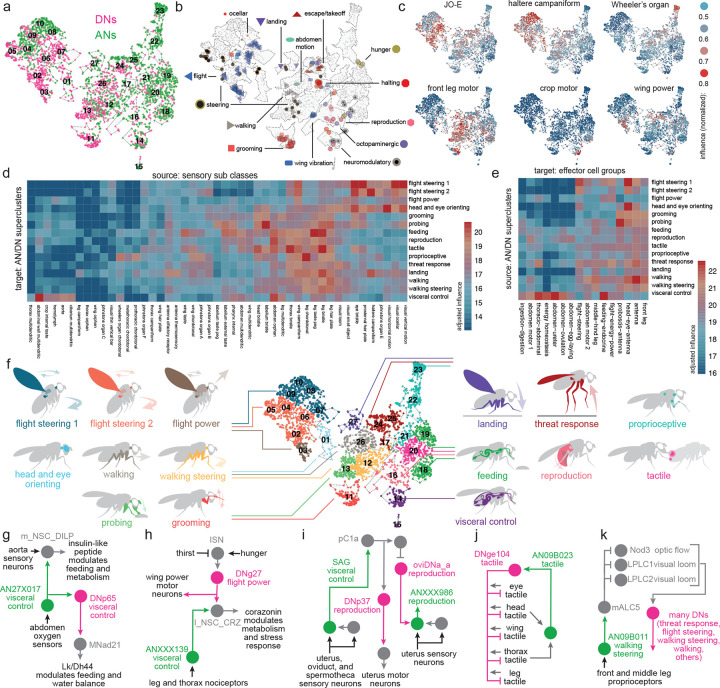
Clustering ANs and DNs into behavior-centric modules. a. UMAP embedding of all ANs and DNs based on cosine similarity between their direct connectivity vectors (connections to any other proofread neuron in BANC). Neuroglancer link to ANs here and DNs here. b. Previously characterized ANs and DNs highlighted in this map (Supplementary Data 7). c. In each copy of this same map, each point is an AN or DN, color-coded by the adjusted influence that cell receives from example sensory neurons (top) or color-coded by the adjusted influence that cell sends to example effector cells (bottom). Based on these adjusted influence scores, we lumped the 27 clusters into 15 superclusters. d. Mean adjusted influence onto each AN/DN supercluster from select groups of sensory neurons. Superclusters are rows; sensory neurons are columns. A subset of visual project neurons were used to determine processed visual streams from the optic lobes^123,137,139–152^, see methods. e. Mean adjusted influence from each supercluster onto select groups of effectors. Superclusters are rows; effectors are columns. f. The same map, here colored by supercluster membership. Neuroglancer link. See (Supplementary Data 4). g. Example circuit involving visceral control ANs and DNs. Neuroglancer link, Codex network. h. Example circuit involving the flight power supercluster and visceral control supercluster. Neuroglancer link, Codex network. i. Example circuit for coordinated visceral sensing and reproductive control. ANXXX986 is female-specific^8,21^. Neuroglancer link. j. Example circuit involving a DN in the tactile supercluster. Neuroglancer link, Codex network. k. Example circuit illustrating proprioceptive input to visual neurons. Neuroglancer link, Codex network.

To identify functional divisions among DNs and ANs, we constructed a map of these neurons based on their direct synaptic connections, both pre- and postsynaptic (Fig. 3a). DNs and ANs are intermingled in this map because, as it turns out, their connections are often similar. We verified that cells with similar known functions are frequently colocalized on this map (Fig. 3b, Extended Data Fig. 3a–c; DNs and ANs with known functions are taken from previous work^9,20,21,24,70,95–126^). We then assessed the influence associated with different AN/DN clusters, considering both influence from sensors and influence onto effectors (Fig. 3c; Extended Data Fig. 3d–f). Based on this information, we grouped related clusters of DNs and ANs into superclusters (Extended Data Fig. 4). Most individual superclusters are influenced by multiple sensory organs (Fig. 3d), and they exert influence onto multiple effector organs (Fig. 3e). Based on these influences, as well as known cells, we were able to link each supercluster with a putative behavior (Fig. 3f).

For example, one supercluster is most likely associated with threat response behaviors. This supercluster contains all the known DNs associated with escape takeoff (Fig. 3b), as well as many DNs and ANs with unknown functions. As a group, these DNs and ANs are influenced by visual loom detectors, visual small object detectors, and specific mechanoreceptors (Fig. 3d). They output to endocrine neurons that regulate internal state, as well as wings and leg motor neurons. All this is consistent with the idea that these DNs and ANs trigger evasive maneuvers, while also recruiting the energy stores needed to support these maneuvers (Fig. 3e).

Another supercluster is most likely involved in reproductive behaviors. As a group, these cells are influenced by tactile sensors, taste sensors, and nociceptors (Fig. 3d). They influence the uterus and reproductive tract, as well as neurohemal complexes, which release signals into the circulatory system (Fig. 3e).

Using a similar process of inference, we linked other superclusters with walking, walking steering, flight steering, flight power, head-and-eye-orienting, grooming, landing, visceral control, feeding, and probing (Fig. 3f). The term “probing” refers to tactile sampling prior to feeding initiation^127^; we propose that this behavior is mediated by the supercluster receiving strong input from labellar tactile afferents and external taste sensors (Fig. 3d), and exerting coordinated influence over the forelegs, proboscis and pharynx (Fig. 3e). Meanwhile, we suggest that a distinct supercluster is associated with feeding: this supercluster receives the highest influence from internal taste sensors (Fig. 3d), and it has strong influence over the pharynx, crop, and salivary glands, as well as endocrine cells targeting the digestive tract (Fig. 3e). The influence of the feeding cluster is strongly correlated with the overall influence of pharynx taste and leg taste receptors (Extended Data Fig. 3f).

The visceral control supercluster contains ANs and DNs that seem to coordinate endocrine cells in different body parts (Fig. 3e). Fig. 3g shows an example circuit involving cells from this supercluster. In this circuit, AN27X017 relays signals from putative abdominal oxygen sensors^58^ (Y.L. and J. T., in preparation) directly to brain endocrine cells that release insulin-like peptide (DILP), which regulates feeding^128^; these ANs converge with the projections of aorta sensory neurons^55^. Meanwhile, AN27X017 also synapses onto DNp65, which targets abdominal leukokinin neurons that regulate feeding and diuresis^129^. This circuit might regulate energy and water balance during physical stress.

Any attempt to put DNs and ANs into categories involves some over-simplification, as many of these cells seem to have multiple functions. Consider, for instance, DNg27, in the flight power supercluster (Fig. 3h). This DN synapses onto wing power motor neurons, as well as brain endocrine neurons that release corazonin (which mobilizes energy stores^58,130^). Thus, this DN is positioned to increase flight power, while also releasing energy needed to sustain flight. Some of the excitatory drive to DNg27 comes from interoceptive neurons in the brain that are suppressed by thirst^118,131^; this connection may help control flight power based on water balance, because high flight power involves high metabolic demand, and thus water loss via respiration^132^. Meanwhile, the same corazonin neurons downstream from DNg27 are postsynaptic to ANXXX139, an AN in the visceral control supercluster that is positioned to relay signals from putative nociceptors. This AN may respond to painful stimuli by recruiting energy reserves, to prepare for struggle or escape. Like many DNs and ANs, these cells are multi-functional.

Within a given supercluster, ANs and DNs can sometimes form extended loops. An example in the reproduction supercluster involves SAG-ANs^111^. The BANC connectome shows that these cells are downstream from sensory neurons in the uterus, oviduct, and spermatheca (Fig. 3i), consistent with their known role as monitors of the reproductive tract^111^. SAG-ANs signal to pC1 cells in the female brain^70,111^, which lie upstream from several DNs in the female reproduction supercluster, including oviDNa_a^70^ and DNp37^133^. DNp37 is positioned to regulate uterine motor neurons^52^, whereas oviDNa_a is positioned to modulate ascending sensory signals from the uterus via interposed ANs (Fig. 3i). Together, these cells form an extended feedback loop linking uterus sensory signals with uterus motor neurons.

We found two superclusters with particularly strong sensory associations: one is dominated by tactile influence, and the other by proprioceptive influence (Fig. 3d). These cells may be involved in whole-body integration of tactile or proprioceptive cues. For example, DNge104 is a cell in the tactile supercluster that is downstream from tactile afferents across the body (Fig. 3j), but also upstream from tactile sensors from those same body parts. Because DNge104 is inhibitory, this circuit could produce tactile contrast enhancement. For example, touching the head or thorax is predicted to excite a specific AN which then increases DNge104 activity, thereby suppressing tactile input to the rest of the body. It is interesting that some DNs and ANs are positioned to primarily influence sensory signals, as targeting a sensory signal can be a powerful way to control a behavior: many sensory neurons will carry a feedback signal to one or more loops, and modulating a feedback signal can cause that loop, in essence, to operate with a different setpoint^53,134,135^.

Even in the behavior-centric superclusters, we can find cells positioned to influence sensory processing. For example, AN09B011 in the walking-steering supercluster (Fig. 3k) makes a strong direct connection onto a visual centrifugal neuron (mALC5), which is positioned to suppress neurons with ventral visual fields, including visual optic flow detectors (LPLC1^136^, Nod3^137^) and loom detectors (LPLC2^138^). This AN is directly postsynaptic to many types of leg proprioceptors, and so it might function to relay leg movement information to mALC5, allowing this circuit to suppress visual responses to leg movement^139^.

In summary, while some AN/DN superclusters seem to specialize in tactile or proprioceptive sensing, most can be associated with a specific behavioral task. This is conceptually analogous to behavior-centric control modules in robotic design^12,13^. Behavior-centric control modules can be useful because they reduce the need for centralized planning and coordination.

### Specialization and coordination among DNs and ANs

**Figure 4:**
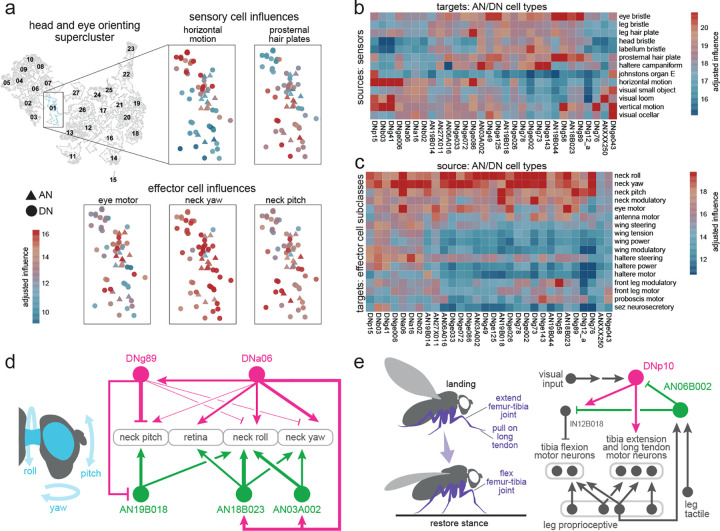
Specializations and coordination within a functional supercluster. a. Enlarged view of the head-and-eye orienting supercluster, taken from the UMAP embedding of all DNs and ANs (Fig. 3d). Top: cells are color-coded by their incoming adjusted influence from two different sensory sources. Same as (a), but now cells are color-coded by their outgoing adjusted influence onto three different effector cell groups. Neuroglancer link, Codex search. b. Mean adjusted influence from sensor sources, for all cell types in the head-and-eye orienting supercluster. c. Mean adjusted influence onto effector cells, for these same ANs and DNs. d. An example circuit with five cell types in the head and eye orienting supercluster. Thick arrows indicate connections with >100 synapses; intermediate arrows indicate connections with 20–100 synapses; thin arrows indicate connections with 5–20 synapses. This example was chosen to illustrate the concept of diverse but overlapping patterns of connectivity within a supercluster, as well as hierarchical interactions between cells in the same supercluster. Neuroglancer link, Codex network. e. An example circuit with two cell types in the landing supercluster (DNp10^100^, AN06B002). This example was chosen to illustrate the concept that ANs and DNs in the same supercluster can be organized into loops. Neuroglancer link, Codex network.

Thus far, we have seen that DNs and ANs can be divided into superclusters. Importantly, the cells in these superclusters are not redundant: their inputs and outputs are specialized. As an illustrative example, consider the head-and-eye-orienting supercluster. Different ANs and DNs in this supercluster are influenced by distinct visual or mechanosensory signals, and they influence different combinations of neck and eye motor neurons (Fig. 4a–c).

Within a supercluster, cells having different specializations are often linked via direct and/or indirect connections. In some cases, particular DNs or ANs are positioned to recruit (or suppress) many other cells in their home supercluster^95^. Again, the head-and-eye-orienting supercluster provides examples of this. For instance, DNa06 is an excitatory DN with connections onto eye motor neurons as well as neck motor neurons that control all three axes of movement (roll, pitch, yaw; Fig. 4d). DNa06 also targets two ANs that are positioned to excite neck and/or eye motor neurons. Meanwhile, DNa06 targets DNg89, which is positioned to inhibit neck-pitch neurons^53^, directly and indirectly through an AN that targets neck-pitch and neck-roll neurons (Fig. 4d). In short, each DN and AN in this circuit is specialized to influence a specific combination of neck and eye motor neurons, and their interactions might serve to coordinate head and eye movements in different directions.

Within a supercluster, specialized ANs and DNs can also be organized into feedback loops. An example of this from the landing supercluster involves DNp10 and AN06B002. DNp10 drives landing maneuvers in response to looming visual stimuli^100^, and we found this cell is positioned to excite tibial extensor motor neurons and also to inhibit tibial flexor motor neurons via an interposed VNC inhibitory interneuron (Fig. 4e), implying that it drives tibia extension during landing. At the same time, we found that AN06B002 is positioned to inhibit DNp10, thereby arresting tibia extension. AN06B002 is postsynaptic to proprioceptive and tactile sensory neurons from the leg (Fig. 4e), and so this circuit motif could form a negative feedback loop that arrests tibia extension when the leg has made contact with the surface during landing, allowing the leg to relax into its normal standing posture as the landing maneuver terminates.

In summary, we find that cells in the same supercluster can have specialized connections to sensors and effectors. For each general behavioral task, there is a set of DNs and ANs that link sensors and effectors in diverse, overlapping combinations. Often, these related cells are interconnected, sometimes in loops. These circuits of finely specialized cells should allow for flexible behavioral control which can be rapidly fine-tuned to the current state of the body and the environment.

### Interactions between behavior-centric modules

**Figure 5:**
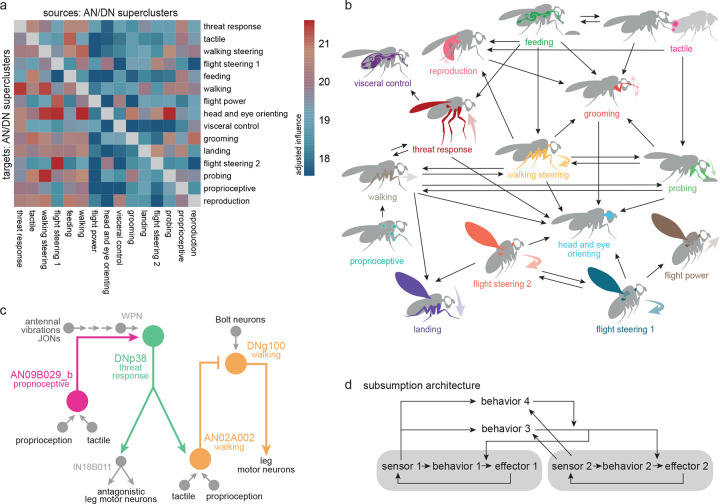
Interactions between behavior-centric modules a. Mean adjusted influence of each AN/DN supercluster on every other supercluster. Values are normalized by the number of cells in each supercluster. b. Summary of the strongest adjusted influences between superclusters. c. A circuit illustrating an example of cross-cluster interactions between DNs and ANs. This circuit links cells in the proprioceptive, threat-response, and walking superclusters. Neuroglancer link, Codex network. d. Schematic example of subsumption architecture. This example has two local loops (behavior 1 and behavior 2), corresponding e.g. the control of individual legs. Behavior 3 is positioned to take control of both local loops (subsumption), contingent on some input from both sensors. Behavior 4 is positioned to subsume all other behaviors, based on some other input from both sensors.

In a system with behavior-centric modules, there should be ways for one module to influence another. In robotic design, this can help prioritize behaviors, resolve conflicts among behavioral drives, and link related behaviors in sequences^12,13^. Indeed, the BANC dataset reveals a specific pattern of influence among AN/DN superclusters (Fig. 5a). Focusing on the strongest of these influences, we can begin to reconstruct relationships between AN/DN behavioral modules (Fig. 5b).

For example, the threat response supercluster strongly influences the walking supercluster (Fig. 5a.b), consistent with the idea that threat responses generally require interruption of ongoing walking. Similarly, flight steering and walking steering strongly influence head-and-eye-orienting (Fig. 5a,b), reflecting the close coupling between head orientation and steering during flight and walking^153,154^. Finally, walking steering influences probing, a behavior that involves pivoting maneuvers where the fly dances around a food source^155^, this interaction might help coordinate proboscis movements with leg movements.

To better understand the circuits that mediate interactions between superclusters, it is useful to drill down to some specific examples. Consider the circuit (Fig. 5c) that involves cells from the proprioceptive supercluster (AN09B029_b), the threat response supercluster (DNp38), and the walking supercluster (DNg100 and AN02A002). Here, AN09B029_b sends ascending mechanosensory signals to DNp38, which also receives antennal mechanosensory signals (via WPNs^156^). DNp38 is positioned to drive co-contraction of antagonistic muscle pairs in all the legs, which would likely increase leg stiffness. Thus, this circuit motif might function to integrate whole-body mechanosensory signals to trigger defensive posture stabilization. Meanwhile, DNp38 is also positioned to recruit AN02A002, which inhibits DNg100, a cell in the walking supercluster downstream from pro-walking Bolt neurons^99^. In this manner, a mechanical threat could stabilize the resting stance while also suppressing walking drive.

Overall, the arrangement of influences between superclusters (Fig. 5b) is conceptually analogous to subsumption architecture in robots (Fig. 5d). In such architecture, some behavior-centric modules are positioned to influence, or “subsume”, another module, in order to exploit its functionality or override it^12,13^. A set of semi-autonomous modules, loosely linked in a subsumption hierarchy, can produce complex, emergent behaviors^13^. This architecture can also potentially account for some hierarchical relationships among animal behaviors^157,158^.

### Linking behavior-centric modules with other divisions of the nervous system

**Extended Data Fig. 5:**
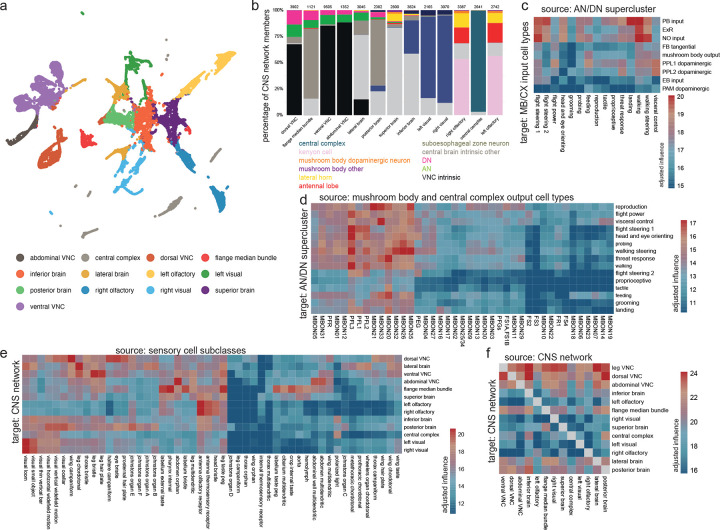
CNS networks’ cluster influence from sensors and to effectors a. UMAP embedding of BANC neurons, where each point is a neuron. This analysis uses all BANC neurons that meet four criteria: they are marked as proofread, they are intrinsic neurons of the CNS (not afferents or efferents), they have >100 incoming and outgoing connections, and no part of the cell is in the optic lobe (as the optic lobes are still undergoing proofreading). In total, 29519 neurons were used for this analysis, corresponding to 88% of cell-typed central brain and/or VNC intrinsic neurons. b. Proportion of each CNS network belonging to select super classes / cell classes. c. Mean adjusted influence of AN/DN superclusters onto input neurons of the mushroom body and central complex. d. Mean adjusted influence of mushroom body output neurons and central complex output neurons onto AN/DN superclusters. e. Mean adjusted influence of sensors onto CNS networks. Visual projection neuron cell types are included, although they are not peripheral sensory neurons. f. Mean adjusted influence of CNS networks onto effector cell subclasses. g. Mean adjusted influence of each CNS network into other CNS networks.

**Figure 6:**
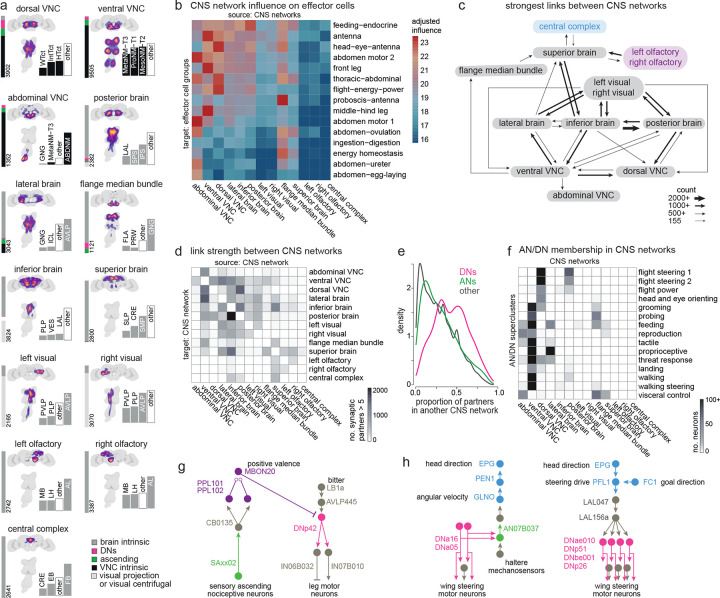
Linking CNS networks with superclusters of ANs and DNs. a. CNS networks, obtained via spectral clustering of 51,502 backbone proofread neurons in the BANC dataset (excluding peripheral neurons and optic lobe neurons but including visual projection neurons and visual centrifugal neurons). Each panel includes a 2D kernel density estimation, a bar plot indicating the network composition, and cell count. Two pairs of networks are mirror images of each other (olfaction right/left and visual right/left), while all other networks are bilaterally symmetric, indicating high bilateral integration in those networks. Anatomical density images are normalized separately for the brain and VNC, based on a random sample of 100k synapses from each CNS network, the hotter the color the denser the synapses. b. Mean adjusted influence of each CNS network on each effector cell group (Fig. 2i). c. Strongest links between CNS networks. The size of each arrow represents the number of postsynaptic cells in that link. One weaker link is shown (155 cells), because this is the strongest output link of the central complex. d. Link strength between CNS networks, measured as the number of postsynaptic cells in that link. The color scale is capped at 2000 cells. e. Out-of-network connections, measured as the proportion of partners each cell has in another CNS network. DNs and ANs have an unusually high proportion of out-of-network connections. The area under each curve is normalized to 1. All three distributions are significantly different from each other (DN vs. other p = 1.92×10^−97^, AN vs. other p = 6.03×10^−5^, AN vs. DN p = 6.74×10^−43^; 2-sample Kolmogorov-Smirnov tests). f. Number of ANs and DNs in each CNS network. ANs and DNs are grouped by supercluster (Fig. 3f). g. Example circuit connecting mushroom body neurons (purple) to ANs and DNs. Neuroglancer link, Codex network. h. Example circuit connecting central complex neurons (blue) to ANs and DNs. Neuroglancer links here and here. Codex network.

Finally, we asked how DNs and ANs are integrated with the rest of the CNS. We began by dividing the CNS into 13 discrete networks, based on each neuron’s direct synaptic connections, using a spectral clustering algorithm that seeks to maximize within-network connectivity while minimizing across-network connectivity (Fig. 6a, Extended Data Fig. 5a). Our aim was to find large groups of interconnected neurons, as these would be candidate coarse functional divisions of the CNS.

Notably, many of these CNS networks contain ANs and DNs (Fig. 6a, Extended Data Fig. 5b). Most CNS networks also have a high influence on effector cells (Fig. 6b, Extended Data Fig. 5a–f). Together, these results suggest that behavioral control is highly distributed across CNS networks. The CNS networks with a high influence on effectors are directly linked in a nearly all-to-all pattern of reciprocal connectivity (Fig. 6c,d, Extended Data Fig. 5f). Interestingly, these links are disproportionately composed of DNs: when we counted each neuron’s synaptic partners outside its assigned network, we found DNs had a relatively high proportion of outside partners (Fig. 6e). We found the same trend for ANs, although this trend was weaker. Most AN/DN superclusters are divided between two or three CNS networks (Fig. 6f), consistent with the notion that ANs and DNs often form bridges between networks. Together, these results argue that ANs and (particularly) DNs have a key role in bridging different functional divisions of the CNS.

Interestingly, the central complex and the olfactory system emerged as networks with distinctive properties. These networks have relatively low influence on effectors (Fig. 6b), weak input from other networks (Fig. 6c,d), and low AN/DN membership (Extended Data Fig. 5b). These networks are likely to have a relatively indirect role in behavioral control: they may merely “supervise” actions, rather than directly controlling actions.

Several example circuits illustrate how these supervisory networks might communicate with lower networks via DNs and ANs (Fig. 6g–h, Extended Data Fig. 5c–d). For example, the BANC dataset shows that putative nociceptive cells in the legs (SNaxx02) project directly to the brain, where they are positioned to excite several mushroom body dopamine neurons, including PPL101 and PPL102 (Fig. 6g). These dopamine neurons encode negative valence^5,159,160^, and they are positioned to instruct olfactory learning in several mushroom body output neurons, including MBON20^5^. Given the synaptic learning rules governing olfactory learning in the mushroom body, we would expect that these dopamine neurons will “teach” MBON20 to respond selectively to odors lacking negative associations -- i.e., odors associated with safety. Notably, MBON20 is positioned to inhibit DNp42, which drives backward walking in response to noxious stimuli^103^. Thus, odors associated with safety should excite MBON20, which is then positioned to suppress avoidance behavior (Fig. 6g). This example circuit illustrates how the olfactory network can supervise behavior by interacting with ANs and DNs.

Another example circuit comes from the central complex, the brain’s navigation center. In the central complex, angular path integration is driven by an internal estimate of the fly’s rotational velocity, encoded by GLNO neurons^161^. The BANC dataset reveals that GLNO neurons receive a strong disynaptic excitatory input from a specific AN (Fig. 6h). This AN receives direct input from DNa16 and DNa05, which likely contribute to steering in flight, via direct and indirect connections onto wing steering motor neurons. Thus, this AN is positioned to send copies of descending flight steering signals back up to the central complex, to update the head direction system in anticipation of an upcoming change in heading. The central complex continuously compares the fly’s estimated head direction against its internal goal direction. This comparison is performed by several cell types, including PFL1^6,162,163^, but the DN targets of PFL1 have not been fully identifiable until now, as DNs were fragmentary in available connectomes. The BANC dataset shows that DNs downstream from PFL1 are in fact putative flight steering neurons (Fig. 6h). Thus, PFL1 is positioned to compare head direction with its goal direction and to generate corrective steering commands in flight when these directions are misaligned. Again, this example illustrates how the central complex can supervise behavior by interacting with ANs and DNs.

## Discussion

The BANC dataset is the first connectome to span the full CNS of a limbed animal. Previous work^7,9–11,14–16^ has used connectome data to analyze the adult fly CNS, but the neurons connecting the brain and VNC were fragmentary in all these datasets^21^, and this limited our ability to connect neurons with behavior. The BANC dataset unifies the brain and VNC for the first time.

The BANC represents a major advance in scale and complexity, compared to other complete connectomes (*C. elegans*^1,2^, *Ciona intestinalis*^4^, and *Platynereis dumerilii*^3^). Tackling a problem of this scale required us to leverage new methods for semi-automated sectioning and EM imaging, computational section alignment, cell segmentation, synapse identification, neurotransmitter assignment, and cell type matching. Because we could draw on the expertise of a large community, we were also able to assemble an embodied connectome with explicit connections to many organ systems.

An embodied connectome of this scale offers new clues about the control architecture of the CNS. In principle, behavioral control could work in a top-down manner, where actions are selected centrally and then relayed to lower regions for implementation, and this has been suggested even for insects^27^. Recently, however, there is new interest in the notion that behavioral control is not centralized, but distributed, in both insects and in vertebrates^29,91,164,165^. Our findings support this latter view. Specifically, our results argue that the core elements of behavioral control are a set of local feedback loops, where effectors are primarily influenced by local sensors. These local loops may be analogous to short feedback loops in the vertebrate spinal cord and brainstem^166,167^. In general terms, local loops are useful because they simplify control and minimize delays. At the same time, purposeful behavior also requires long-range coordination among body parts, and this is mediated, in part, by DNs and ANs. The BANC dataset allowed us to systematically analyze *Drosophila* DNs and ANs for the first time. We found these cells could be divided into superclusters, with each supercluster linking a specific set of sensory cells and effector cells. Moreover, we found that DNs and ANs organize effector cells into discrete clusters of co-regulated motor/endocrine units. We were able to link many AN/DN superclusters with putative behavioral functions, reminiscent of behavior-centric control modules in robotic architecture^13^.

The gap between the brain and the VNC is often called a bottleneck of information transfer within the CNS^19,21^, but in fact, the sheer number of DNs (~1300 cells) and ANs (~2400 cells) is much larger than the number of effector cells in the BANC dataset (~1000). If we think of DNs and ANs as “wires” for actuating effector cells in different combinations, then the large number of DNs and ANs suggests that effector cells can be actuated in many different combinations. Indeed, within each AN/DN supercluster, we find many fine-grained variations on the same connection pattern, forming parallel pathways with slightly different inputs and/or outputs. This arrangement should promote flexibility, by offering many available action patterns. It should also promote precision, by pre-selecting the specialized action patterns that can result from particular patterns of sensory input. These sorts of connectivity specializations could explain why, for example, different threat response DNs can produce different escape takeoff maneuvers^168^, and why different walking-steering DNs can produce distinct changes in leg movement^105^.

Finally, when we analyzed the network structure of the entire CNS, we found that the links between different networks are enriched for ANs and (particularly) DNs. Importantly, we found that many CNS networks have a high influence on effectors, supporting the idea that behavioral control is distributed, rather than centralized. We found that a few CNS networks -- particularly the central complex and the olfactory system -- have a relatively low influence on effectors, suggesting these networks have a supervisory role, rather than a direct role in behavioral control. This type of supervision is characteristic of subsumption architecture in robotic design, where high-level modules have the ability to recruit or suppress lower-level modules, but these high-level modules are not actually required for any but the most complex behaviors^12,13^. In the future, it will be interesting to investigate why supervisory networks like the central complex can have such profound behavioral effects^169,170^, given their weak anatomical connection to effector cells.

This project illustrates how insight can arise from new technologies, combined with the accumulation of many small biological facts. Just as early cartographers amalgamated the work of other map-makers, we have deliberately amalgamated typology and metadata from prior *Drosophila* connectomes. The workflow we developed is conceptually similar to the workflow that amalgamates information from emerging genomes. The BANC is a living public dataset which should progressively improve as long as users continue to interact with it. This open science effort should generate even more testable experimental hypotheses and, ultimately, new theories.

## Methods

### Specimen

The Brain and Nerve Cord (BANC) sample came from a female adult fly. We behaviorally screened 5–6 day post-eclosion wild-type *Drosophila melanogaster* (F1 progeny of a w^1118^ × Canton-S cross) female flies^171,172^. The fly used for the BANC dataset turned right 70% of the time over 582 choices when walking in an acrylic Y-maze for 2 hours. We raised the flies on standard cornmeal-dextrose medium at room temperature (~20 °C) in natural lighting conditions. We collected flies on the day after eclosion, housed them in vials with other flies for 4–5 days, behaviorally tested them and then subsequently housed them individually in vials for ~1 day at 25°C until dissection.

To dissect the flies, we pinned them individually onto a dissection pad then submerged them in a drop of ice cold Karnovsky's fixative (2.5% formaldehyde, 2.5% glutaraldehyde in 0.1M cacodylate buffer, pH 7.4) containing 0.04% CaCl_2_. We removed the legs and proboscis removed to allow fixative to access the nervous tissue. Next, we carefully removed the head capsule and the cuticle of the ventral thorax to expose the nervous tissue for dissection. Within 5 minutes, we completely dissected the brain and connected VNC, and we transferred it to an Eppendorf tube containing the same Karnovsky's fixative. We fixed the sample at 4 °C overnight. On the subsequent day, we washed the sample with 0.02M 3-amino-1,2,4-triazole (A-TRA) in cacodylate buffer (3×10min) and then we stained it with 1% OsO_4_ in 0.1M A-TRA for 90 minutes on ice. On the same day, we stained the sample with 1% thiocarbohydrazide for 8 minutes at 40 °C, 2% OsO_4_ (aqueous) at room temperature for 60 minutes, and 1% uranyl acetate in maleate buffer at 4 °C overnight. On the next day, the sample was stained with lead aspartate for 3 hours at 60 °C, then dehydrated in a graded ethanol series, washed with propylene oxide, and infiltrated with 2:1 and 1:2 propylene oxide:LX-112 resin consecutively for 30 minutes each. The sample was then placed in pure LX-112 resin overnight at 4 °C and was embedded in fresh pure resin the following day and cured at 60 °C for 48 hours.

The resin-embedded sample was scanned on a microCT X-ray scanner (Zeiss) before serial sectioning to screen for obvious defects or damage. Importantly, the neck connective appeared intact. The specimen includes the central brain, neck connective, VNC and the medulla, lobula and lobula plate of the optic lobes. It lacks the lamina (part of the optic lobes), the ocelli and the ocellar ganglion. Thus, the R1–6 photoreceptors and the ocellar photoreceptors are missing from BANC (~10000 cells) and intrinsic neurons that arborize in the lamina and ocellar ganglion are incomplete (cell types: L1–5, Lai, T1, C2, C3, Lat, Lawf1, Lawf2, OCG01, OCG02, OCC01, OCC02, DNp28 and the ocellar local neurons). The BANC is the only available dataset for which the complete female abdominal neuromere is available.

### Serial sectioning

We cut serial 45–50 nm thin sections and collected them on a 7500-slot reel of GridTape (Luxel) as previously described in^7^.

### Transmission electron microscopy (TEM) imaging

We used one TEM (JEOL 1200 EX) with a custom vacuum extension and scintillator (Grant Scientific), 2 × 2 array of sCMOS cameras (Andor, Zyla 4.2), and custom modified with a reel-to-reel, GridTape imaging stage to acquire the dataset as described previously^7^. Imaging spanned 7.5 calendar months, but 96.5% of the images were acquired during the 4 months of November 2021 to February 2022.

### Missing data

Of the 7010 sections, 6970 (99.43%) were collected and imaged without data loss. Ten (0.14%) have no data due to the section being lost (sections 856, 885, 3755, 5746, 5772, 5778, 5793, 5801, 5822 and 5869). Notably none of the losses are consecutive serial sections. One of these losses (3755) was because the section was collected onto the wrong location on the GridTape (not over the slot) and so it could not be imaged with TEM. The other 9 losses were due to the support film rupturing after section collection but before the section could be imaged. An additional 30 sections (0.43%) have partial data: 11 sections are missing all images for the brain: 914, 1462, 5841, 5849, 5888, 5896, 5916, 6207, 6208, 6209 and 6210; 7 sections are missing all images for the VNC: 874, 2784, 2822, 3064, 3102, 4566 and 5840; 12 sections are missing a fraction of brain and/or VNC images: 2828, 2860, 2912, 2986, 3054, 3080, 3586, 3605, 3833, 4648, 4768 and 5935. The large majority of these losses were also caused by partial rupturing of the support film before the tissue was imaged.

### TEM dataset alignment and segmentation

We performed initial BANC image alignment with a custom software pipeline that deployed AlignTK alignment functions (https://mmbios.pitt.edu/aligntk-home) on a computing cluster^7^. We refined the alignment of the data using self-supervised CNNs and online optimization to produce displacement fields that were combined with a global relaxation^173,174^. We next trained a CNN to identify regions that were damaged during serial sectioning. We then used CNNs to segment the dataset into cells and fragments of cells at 16 × 16 × 45 nm^3^, excluding regions that decreased cell segmentation performance including areas with damage, as well as organelles including nuclei and mitochondria^8,31^. We then ingested the automated segmentation into the Connectome Annotation Versioning Engine (CAVE)^33^ for distributed proofreading.

### Synapse detection

We generated synapses in two-steps: (1) postsynaptic terminal detection and (2) synaptic partner assignment^175^. We pretrained both models with data from FAFB, and we tuned the detection model with additional labels from the BANC. The detection operated on 8 × 8 × 45 nm^3^ images, with an output at 16 × 16 × 45 nm^3^. We removed detection objects <3 voxels. Assignment operated at 16 × 16 × 45 nm^3^. We merged terminals with identical assignments that were within 200 nm of each other into a single terminal. This detection is known as synapses_250226 and is available through CAVE. It comprises 218460852 synaptic links, of which 65% of presynaptic ends and 22% of postsynaptic ends are connected to a proofread neuron.

### Synapse prediction evaluation

To determine the false-positive rate of the synapse detection, we randomly selected 1000 synapses from across the dataset (~70 synapses per neuropil region and for all of the nerves combined, total: 4648) and manually scored them as true synapses, ambiguous, or false positives (Extended Data Fig. 1c). We also evaluated synapses on a 2 × 2 × 2 μm^3^ cutout from the mushroom body, a known problem area for our detection method: F-score: .79, Precision: .68, Recall: .95. Because this detection relies on identifying postsynaptic profiles, some classes of synaptic connection for which postsynaptic sites are less distinct may be under-detected. We know that our average number of outgoing connections for Kenyon cells (139) is far smaller than in FAFB (213, cleft score threshold > 50). Another area of under-detection may be axo-axonic connections between sensory neurons. The BANC detection has an autapse rate of 2.1%, a majority of which we expect to be a misassignment of the presynaptic link from a correctly detected postsynaptic link. We recommend users filter out autapses in their analyses.

### Neurotransmitter prediction

We used a recently described approach to predict neurotransmitter type at each automatically predicted synapse^46^. Briefly, we trained a 3D convolutional neural network (CNN) to classify presynapses into one of eight neurotransmitter classes: acetylcholine, dopamine, GABA, glutamate, histamine, octopamine, serotonin, or tyramine. We compiled ground truth data for synaptic transmission from the literature^6,9,60,61,63,64,67,70,118,120,121,130,133,137,140,176–255^, totaling 4545 identified cell types from FAFB/MANC/Hemibrain. Of these, members of 2930 cell types (37878 neurons) could be found in BANC. We removed motor neurons from the ground truth, as they have few presynapses within the CNS. The complete dataset was split by neuron into training and testing sets, with 80% of the data for training and the remaining 20% for testing. This resulted in 16448 neurons for training and 4124 for testing. We used the following sampling strategy to ensure a balanced dataset across different neuron types. For neurons associated with the most common neurotransmitters (acetylcholine, GABA and glutamate), we randomly sampled a maximum of 10 presynaptic sites from each neuron. For all other neurotransmitters, we included all identified presynaptic sites. This approach ensured that all cell types that had ground-truth were represented in both training and testing sets. The input data for the network consisted of 3D cutouts from the EM volume, each centered on a presynaptic site. These local cutouts had dimensions of 640 × 640 × 630 nm. We used a 3D CNN architecture based on the 18-layer residual network (ResNet-18)^256^. ResNet-18 includes 3D convolutional layers, batch normalization and ReLU activation functions, with the core of the architecture consisting of residual blocks that use skip connections to enable effective training. The model architecture was adapted for our task by modifying the initial convolutional block to accept single-channel grayscale input from EM data. Finally, we replaced the model’s original fully-connected output layer with a linear layer that maps the learned features to our eight specific neurotransmitter classes, followed by a softmax activation to produce the final probability distribution. The network was trained using the Adam optimizer^257^ to minimize the focal loss function^258^. This loss function is a variant of the standard cross-entropy loss, which is effective for datasets with a significant class imbalance as it down-weights the loss assigned to well classified samples, allowing the model to focus on difficult-to-classify samples. To further improve generalization of the model, we applied several data augmentation techniques during training. These included random affine transformations, random noise, and random gamma correction. The probability of applying these augmentations was increased for less frequent neurotransmitter classes to further mitigate the class imbalance. We trained the model for 1,060,000 iterations using a batch size of 16 samples. The final model selected was the one that achieved the highest classification accuracy on the separate testing set. A neuron-level transmitter prediction is obtained by summing the classification probabilities for each predicted class across all presynaptic detections, and selecting the class with the highest total confidence as the most likely neurotransmitter; we assume Dale’s law^259^ holds even though we know that an unknown proportion of neurons in the CNS co-transmit with multiple fast-acting transmitters^46,190,192,260^. Though marginally improved, as in^46^, we expect a large proportion of our serotonin predictions in particular to be incorrect, as the network seems to guess serotonin for peptidergic neurons that lack clear signs for another classification. A fully cited compilation of ground truth labels per cell type can be found here: https://github.com/funkelab/drosophila_neurotransmitters/tree/main, collated by A.S.B., D-Y.A. and J.F.

### Neuropils and template alignment

To transform the BANC data into a standard template space for analysis and inter-dataset comparisons, we computationally generated a ‘neuropil stain’ based on the synapse prediction^175^. To do this, we downsampled and Gaussian blurred (σ = ~900 nm) the predicted synapse locations to produce a synapse density map at the approximate resolution of light microscopy data used in the Drosophila standard templates. We then registered the synapse density map of the EM dataset to the JRC 2018 Female brain and JRC 2018 Female VNC templates^261^ separately using elastix (https://elastix.lumc.nl/). Leveraging this alignment, neuropils and neurons were transformed between different connectome datasets for visualization and quantitative comparison in the same coordinate system. Meshes for individual neuropils in the central brain^35^ and VNC^36^ were based on previous work. We generated a left-right registration for BANC based on a thinplate-spline warping registration built from matched points on identified pairs of ~30 DNs, available through the bancr R package.

### Proofreading

We proofread neurons to correct automated cell segmentation errors as we described previously^15^. Members of our respective laboratories, dedicated proofreading teams at Princeton, SixEleven (Davao City, Philippines), and Aelysia (Bristol, United Kingdom), as well as a community of citizen scientists collaboratively undertook this effort. We used a multi-pronged strategy. To capture neurons with cell bodies in the CNS, we proofread segments associated with automatically-detected nuclei, which were then extended to reconstruct their full morphology and remove false mergers. To include sensory neurons, whose cell bodies typically reside outside the CNS, we seeded every neuron profile in planes that cut a cross-section through a nerve (1 plane per nerve, except in cases where 1 plane could not capture the full cross-section of the nerve; 47 seed planes total) and then reconstructed starting from those seeds. To capture all neurons in the neck connective, we seeded two planes that were cross-sections through the neck connective (y = 92500 and y = 121000). These transverse planes were positioned posterior to the central brain and anterior to the VNC. Additionally, we proofread orphan segments containing >100 presynaptic links in decreasing order of synapse count for the central brain and VNC. We considered a neuron ‘backbone proofread’ when its primary neurites (if not sensory), or major microtubule-rich processes had undergone a thorough review^32^. This indicated that we expected the overall morphology of the cell to be correct and that, while minor branches or a small number of synapses might still require adjustment, we did not anticipate future proofreading to radically alter the neuron's core shape or identity. We proofread 114,610 neurons to ‘backbone proofread’. In total, 155 people served as proofreaders for the project (defined as people who made ≥100 edits).

### Color MIPs

We generated color-depth maximum intensity projections (colorMIPs) of all proofread neurons using the BANC python package (https://pypi.org/project/banc/). We registered neuronal reconstructions to JRC2018_Unisex_20x_HR (1210×566 px) and/or JRC2018_VNC_Unisex_40x_DS (573×1119 px), for compatibility with NeuronBridge^262^.

### Cell-type matching and annotation

#### Overview

Previous studies have invested substantial effort in cell typing both the brain^5,6,14,17,41^ and VNC^7,8,10^, employing a combination of manual annotation and computational methods. Our approach leverages morphology and connectivity matching to cell type the ~160,000 neurons in the BANC dataset by associating them with published reconstructions, namely FlyWire-FAFB v783^15^ and MANC v1.2.1^11^. We have successfully assigned cell type labels to 53% of BANC neurons (82813 neurons, 74% excluding the optic lobes), with an estimated error rate of ~7% based on sampling 1,000 matched neurons. The mismatched neuron was almost always a similar cell type within the same hemilineage. For the remaining neurons that could not be confidently matched, we have classified them based on gross morphology and identified their closest associated neurons in other datasets with NBLAST. We estimate that ~10% of these unmatched neurons will prove unmatchable due to reconstruction quality issues or developmental differences in neuron wiring. Notably, we estimate that as many as 1177 neurons of the VNC may be sexually dimorphic and cannot be matched well to MANC (which is a VNC sample from a male fly). Our VNC annotation work (A.M, C.K.S et al., in prep) significantly advances connectome analysis by enabling direct comparisons with established identified cell types in the field and facilitating integration with existing datasets, particularly FAFB and MANC, which can be done with FlyWire Codex.

#### Process

Using NBLAST^39^, which quantifies pairwise neuronal similarity by considering both the position and morphology of neuronal arbors and calculating similarity scores by comparing matched morphological segments, we automatically identified potential matches between BANC neurons and those FlyWire-FAFB v783^15,17^ and MANC v1.2.1^10^. Following automated NBLAST scoring, we manually reviewed candidate matches. For sensory neurons, ANs and DNs and intrinsic neurons of the brain, this manual review involved co-visualizing the meshes of matched neurons in 3 orthogonal 2D projections and evaluating the correspondence. For ANs and DNs, we followed up this 2D comparison with co-visualization and manual evaluation in 3D using neuroglancer. For intrinsic neurons of the VNC, we also used connectivity to automatically determine their similarity to MANC neurons. When the top matched cell type agreed between NBLAST and connectivity, we assigned the neuron to that cell type; when these potential matches were in conflict, we co-visualized the BANC and MANC neurons in 3D in neuroglancer and manually reviewed them to determine the correct cell type. High NBLAST scores (e.g., above 0.3) generally indicated a strong likelihood of a correct match. Iterative proofreading and matching increased the population of identified cells as sometimes, low NBLAST scores indicated issues with neuron reconstruction, which suggested additional proofreading was necessary.

For many afferent and efferent neurons, in addition to matching to FAFB and MANC, we used comparisons to the literature and the domain expertise of our authors to determine their cell types and functions. In particular, we identified leg and wing motor neurons by their morphology and connectivity, as previously described^8^. The key identifying features we used were the exit nerve of the axon, the relative trajectory of the primary neurite, the relative position of the soma, and unique features of the dendritic morphology. Front, middle, and hind limb neuropils differ in terms of specific morphology yet the identifying motor neuron features largely retain their relationships, allowing us to identify homologous motor neurons in each neuropil^9^. We confirmed morphological identification by comparing these motor neurons on the basis of the sources of common synaptic input^8^. We identified endocrine neurons of the brain based on morphology and the cosine similarity of their connectivity with each other and with the FAFB endocrine neurons. We used morphological comparisons to the literature to identify the motor neurons of the antennae, eyes, neck, crop, haltere, pharynx, proboscis, pharynx, salivary glands and uterus; octopaminergic effector neurons involved in ovulation; endocrine neurons of the VNC; and chemosensory, tactile and proprioceptive sensory neurons from the head, eyes, antennae, proboscis, legs, abdomen, wings and halteres^92^. In some cases, we used data from the larval fly (putative nociceptive, putative oxygenation and aorta sensory neurons^10,55,57,58,263–266^) to annotate suspected homologous neurons. Adult nociceptors will be reported (J.J. & J.C.T., in preparation). We subjected chordotonal, campaniform and hair plate neurons of the VNC, including those of Wheeler’s organ, the prothoracic organ and the metathoracic organ, to additional careful review and re-annotation^7,73,267–269^.

#### Neurons of the neck connective

We reviewed all profiles in the two seed planes through the neck connective. We successfully proofread 98.3% of the neuronal profiles to ‘backbone proofread’ status, for a total of 3695 proofread neurons. We then matched these neurons to cell types in FAFB and MANC, as described above. We identified 1841 ANs, of which we matched 1725 (corresponding to 538 cell types), and 1313 DNs, of which we matched 1288 (corresponding to 474 cell types). In addition, we identified 13 sensory DNs (afferent axons that enter through a brain nerve and project through the neck connective to the VNC, discussed in more detail here^21^) corresponding to 5 cell types, 511 sensory ANs (afferent axons that enter a VNC nerve and project through the neck connective to the brain) corresponding to 39 cell types and 5 efferent ANs (ANs that also project out of other nerves) corresponding to 3 cell types, including EAXXX079, which may be the leucokinin ANs in^270^. For ANs, sensory ANs and efferent ANs, we use the MANC cell type name; for DNs and sensory DNs, we use the FAFB name. When this resulted in the same name for different cell types (which became apparent when considering the full neuron rather than just the brain or VNC half), we appended an underscore and a letter to the FAFB/MANC name. We also identified and proofread 49 efferent neurons of the neck that leave through the cervical nerve. These are neck motor neurons, and we named them as in^53^. Note that because they do not traverse the entire extent of the neck connective, they are not included in our count of 3695 “backbone proofread” neurons of the neck connective. We do not use sensory or efferent ANs and DNs in our analysis of ANs and DNs. In our review of the neck connective, we identified 31 ANs and DNs that appeared to have developed abnormally or were stochastic in whether they had an ascending/descending arbor. For example, DNge079 on the right-side (in MANC named DNxl080) has a mis-targeted dendrite located in the VNC, rather than the central brain. However, we note that both the left and right IN08B003 neurons are ANs in this dataset but are intrinsic neurons of the VNC in MANC and in FANC. We determined that the cell type DNg28 leaves the brain through the maxillary-labial nerve and after it re-enters through the same nerve, its processes remain outside of the glial sheath surrounding the CNS as it then traverses the neck to envelop the outside of the VNC and target neurohemal release sites. Therefore, we re-classified it from a DN to solely an efferent cell type. As in FAFB, we could not find DNg25, and DNd01 was not a DN but rather a central brain intrinsic neuron^21^. Important prior work bridged a proportion of ANs and DNs between FAFB and MANC using available experimental data^21^, which was a valuable resource of our matching efforts.

#### Annotation taxonomy

We annotated neurons hierarchically by flow (afferent, intrinsic, efferent), super class (eg. sensory, motor, visceral/circulatory, ascending, descending), cell class (eg. chordotonal organ neuron, leg motor neuron, kenyon cell), cell subclass (eg. wing steering motor neuron, front leg hair plate neuron, PPL1 dopaminergic neuron), individual cell type, and with associated metadata (region, side, nerve, body part sensory, body part effector, peripheral target type, cell function, cell function detailed, hemilineage, neurotransmitter verified, neuropeptide verified, FAFB v783 match ID, MANC v1.2.1 match ID and other names). The full list of terms used in each category are listed in Supplementary Data 1. This framework enabled both broad and fine-grained categorization, such as distinguishing different and specific classes of sensory neurons. We imported annotations from cell type matching to existing *Drosophila* connectomes^10,15,17^ as well as those that proofreaders and the community contributed through a custom Slackbot (https://github.com/jasper-tms/the-BANC-fly-connectome/blob/main/slackbots/annotation_bot.py) directly to CAVE, facilitating real-time tagging and collaborative refinement. We updated annotations as proofreading progressed, and they are publicly available through FlyWire Codex and on CAVE (cell_info and codex_annotations tables).

#### Influence

The influence score^271^ quantifies the influence of the activity of a neuron or group of neurons, called the seed, on each of the other neurons in the network. It is a measure of steady-state activity, resulting from continuous stimulation of seed neurons. We compute steady-state activity assuming a linear dynamical model of neural activity,

$$\tau \frac{dr(t)}{dt}=-r(t)+Wr(t)+s(t)$$

where *r* is the vector of neural activity, *W* is the connectivity matrix, τ is the network time constant, and *s* is the simulated neural stimulation. For each seed, all elements in *s* corresponding to the seeded neurons are set to one, while the remaining elements are fixed at zero.

The weight of each connection is taken as the number of synapses in that connection, normalized by the total count of input synapses onto the postsynaptic cell in question. That is, if *c*_*ij*_ is the synapse count from presynaptic neuron *j* onto postsynaptic neuron *i*, then the total input count for neuron *i* is $N_{i}=\underset{j}{\sum }c_{ij}$, and the connectivity weights were set to *w*_*ij*_ = *c*_*ij*_/*N*_*i*_. This type of normalization follows previous work and has been shown to qualitatively capture experimental observations^41,272^. All connectivity weights are treated as nonnegative values, because our goal was to generate a proxy for the number of ‘hops’ in a connection, and previous synaptic hop metrics have been unsigned^17,22^; moreover, the signs of many connections are still unknown. To ensure stable neural dynamics, we re-scaled *W* such that its largest real eigenvalue is 0.99.

We compute the steady-state solution for the assumed network dynamics by

$$r_{\infty }=-{\left(W-I\right)}^{-1}s,$$

separately for each seed vector *s*. As *W* is a highly sparse matrix, we could compute this solution efficiently using the sparse matrix parallel computing libraries PETSc and SLEPc (https://petsc.org/release/ and https://slepc.upv.es/). If the seed is one cell, and we are interested in a single target cell, we simply take the steady-state activity of the target *r* in response to the seed. We define *r*_*ij*_ as the steady-state response of target cell *j*, given stimulation of seed cell *i*. Often, we are interested in pools of related target cells (e.g., a pool of related motor neurons). Thus, for a target pool *T* that contains the indices of the |*T*| = *N* target neurons, we take the average steady-state response of each cell in the target pool, $\bar{r_{T}}=\frac{1}{N}\underset{j\in T}{\sum }r_{ij}$. Similarly, we are often interested in a pool *S* of related seed cells, where *S* contains the seed cells’ indices. Here, we could simulate activity in all seeds individually, and average the results. In this case, for a seed pool of size |*S*| = *M*, the average response is

$$\bar{r_{T,S}}=\frac{1}{NM}\underset{i\in S,j\in T}{\sum }r_{ij}$$

Alternatively, because the steady-state solution *r*_∞_ is linear in the seed vector, it is sometimes more convenient to just simulate activity in all seed cells simultaneously. In this case, if *r*_*j*_ is the response of the *j*th target cell to the simultaneous activity of all seed cells, we take $\bar{r_{T}}=\frac{1}{NM}\underset{j\in T}{\sum }r_{j}$.

In this type of simulated network, $\bar{r}$ will generally decay exponentially as the distance increases between the seed and the target (in network space). To correct for this, we take the logarithm of $\bar{r}$. And because $\text{log}(\bar{r})$ is generally negative, we add a constant *c* that brings the values of $\text{log}(\bar{r})$ into the nonnegative range, for ease of display. The resulting value is called the “adjusted influence”:

$$\text{adjusted}\;\text{influence}=\text{log}(\bar{r})+c$$

We used *c* = 24, because his ensured that all adjusted influence values were non-negative (given that -24 was approximately the minimum value of $\text{log}(\bar{r})$ we observed). Across the entire CNS, a small and discrete group of cells had $\text{log}(\bar{r})\ll -24$ for any seed, as these cells were not well-connected to the graph; we set these adjusted influence values to 0.

We confirmed that adjusted influence is proportional to the number of synaptic ‘hops’ separating the seed cells and target cells, as expected, and this was true for two different published metrics of hops length (Fig. 2b. and Extended Data Fig. 2a; see below for details of these previous metrics). Thus, adjusted influence is essentially a computationally efficient and deterministic method of estimating the effective number of hops separating the seed and the target. Because the number of hops is an unsigned quantity^17,22^, it is reasonable that adjusted influence is also unsigned. As compared to previous metrics of hop number, adjusted influence has several advantages. First, we have an explicit expression for the steady-state solution, making the computation more efficient relative to comparable activity propagation approaches^15,22,41^. Second, the steady-state solution is linear in the seed vector, such that it can in principle be summed across different seeds.

Rather than taking the steady-state activity as the basis for this influence metric, we also considered using the initial slope of the neural activity. However, the initial slope turned out to be directly proportional to the chosen seed vector, which made it unsuitable as a measure to quantify network-wide influences. We furthermore considered projections of the above dynamics into the top 1000 eigencircuits, similar to previous work^82^, but we found this truncation to be unsuitable for our purposes to well-approximate the full network dynamics.

We computed the influence scores reported in this paper using Python 3.13.2, and we executed all computations using a MacBook Pro running macOS Monterey version 12.6.9. The code used to compute the influence scores is available as a separate Python package (see ‘Code availability’ section).

#### Alternative metrics of polysynaptic connectivity

For comparison with our influence scores, we used two complementary probabilistic graph traversal algorithms to model information flow through the CNS. First, we applied the signal cascade approach^22^, in which activity propagates from a set of seed neurons to downstream targets based on synapse counts, treated as proxies for synaptic strength. A key feature of this model is that neurons are activated only once and then enter a deactivated state, enabling assessment of potential temporal sequences of activation.

Second, we used an information flow model^15,41^, in which neurons are probabilistically recruited based on the fraction of synapses received from already recruited neurons. This model allows ongoing activation from previously active neurons and assigns each neuron a rank that reflects its integration point in the circuit. While these ranks do not correspond to true physiological latency, this approach enables systematic inference of information flow directionality and network layering across the CNS.

#### Spectral clustering

We adapted a spectral clustering algorithm^273^ to partition the CNS into modules of highly interconnected cells. For this analysis, we focused on intrinsic neurons of the central brain and VNC, ANs, DNs, visual projection neurons, and visual centrifugal neurons. (We chose to exclude optic lobe neurons because they are so numerous that they end up dominating the analysis.) Starting with these 42,639 cells, we iteratively pruned cells that did not have at least one input and output partner among the remaining cells (e.g. because all their input comes from sensory neurons, or all their output goes to motor neurons, etc.). This left 41,951 cells as the input to this analysis.

To apply spectral clustering, we first specified our population of *N* cells of interest and a desired number of clusters *k*. We then constructed a weighted, undirected graph whose nodes corresponded to these *N* cells and whose edge weights were derived from the connectome. More formally, edge {*i*, *j*} was assigned weight

$$a_{ij}=\frac{1}{2}\left(w_{ij}+w_{ji}\right)$$

where *w*_*ij*_ is the normalized synaptic input from presynaptic cell *j* to postsynaptic cell *i*, as defined above. We then computed the first *k* eigenvectors of the graph Laplacian, which resulted in a *k* × *N* matrix of unit-norm eigenvectors *X*. Each node then received a *k*-dimensional feature vector that was determined by its loadings onto the eigenvectors, yielding an *N* × *k* feature matrix *Y* with entries

$$y_{im}=\frac{x_{mi}}{\sqrt{\underset{m}{\sum }x_{mi}^{2}}}.$$

Finally, we applied k-means clustering to these feature vectors to assign each node to a cluster. We decided to use 13 clusters because this produced a coarse-grained division at the approximate level of resolution we found relevant to our analysis, and also because the resulting cluster divisions largely corresponded to salient boundaries in the UMAP space of CNS neurons.

### Data analysis

Visual projection neuron functions were used to account for different visual information streams as ‘sensors’. This is an incomplete survey of visual functions bounded by the literature^123,137,139–152^. We used for visual_chromatic - aMe12, MeTu3b, MeTu3c, MTe50; visual leg feedback - LT52; visual horizontal wide field motion - dCH, FD1, FD3, H1, LPT04_HST, LPT21, LPT22, LPT23, LPT26, LPT42_Nod4, Nod1, Nod2, Nod3, vCH; visual large_objects and visual thin vertical bar - LC15; visual loom - LC16, LC4, LPLC1; visual object and visual loom - LC12, LC17; visual polarized light - MeMe_e10, MeTu2a, MeTu2b, MeTu3a; visual small_object - LC10a, LC10b, LC10c, LC10d, LC11, LC13, LC18, LC21; visual small_object,visual_loom - LC26, LC6, LC9, LPLC2; visual thin vertical bar - LC25, MeTu1; visual vertical wide field motion - LPT27, LPT28, LPT30, LPT31, LPT45_dCal1, LPT47 vCal2, LPT48 vCal3, LPT49, LPT50, Nod5, V1, vCal1, VST1, VST2. Sensory neuron cell functions were determined by a literature search and search of extant connectome meta data, for information on their peripheral sensory organs/structures. Through this manuscript, we clustered heatmaps using hierarchical clustering based on Ward’s distance using functions from base R. We applied dynamic tree cut^274^ (implemented as dynamicTreeCut::cutreeDynamic, using deepSplit = 4) clustering to UMAPs to delineate effector and AN/DN clusters, other than in Fig. 6 and Extended Data Fig. 5, in which spectral clustering was used, see above. We conducted data analysis in R using the uwot^275^, tidyverse^276^ and ggplot2^277^ packages. We made the Kernel density estimates for Fig. 6a using MASS::kde2d, n=100, cubes with densities above the first percentile colored^278^. We calculated cosine similarity using the lsa R package^279^, and we applied it to direct connectivity between BANC neurons to build the space used in Fig. 3. To perform the Kolmogorov-Smirnov test in Fig. 6e, we used the kstest2 function in MATLAB 2024a (Mathworks). We used LLM assistance to review and recommend code as well as to draft code documentation, all of which we consciously evaluated for accuracy and which was in compliance with the Harvard University Generative AI guidelines (https://www.huit.harvard.edu/ai/guidelines). The Harvard AI Sandbox (https://www.huit.harvard.edu/ai-sandbox) provides a secure environment in which to use LLMs, and all queries are recorded. The majority of our codebase was not assisted by LLMs.

## Extended Data

## Supplementary Material

Supplement 1 Supplementary Data 1: Annotations taxonomy
Table of categories of annotations applied to BANC neurons and the list of terms used in each category. For region, side, flow, super_class, cell_class, cell_sub_class, cell_type, and hemilineage, only one term applies per neuron. For the other categories, neurons can be labeled with more than one term.
flow - from the perspective of the whole CNS, whether the neuron is afferent, efferent or intrinsicsuper_class - coarse division, hierarchical below flowcell_class - hierarchical below super_classcell_sub_class - hierarchical below cell_classcell_type - the name of the matched neuron from FAFB if it is a brain neuron or a DN or the name of the matched neuron from MANC if it is a VNC neuron or an AN. There are a few exceptions where those names did not define single cell types and were further split. This is hierarchical below cell_sub_classregion - region of the CNS; all neurons that have arbors in the optic lobe are considered optic_lobe and all neurons that fully transit the neck connective between the brain and VNC are considered neck_connectiveside - from the fly’s perspective, the side on which the cell body is located or for afferent neurons, the side of the entry nerve.cell_function - term briefly describing the function of the neuron, applied largely to afferent and efferent neuronscell_function_detailed - more detailed information for the function of the neuron than cell_function, also applied largely to afferent and efferent neuronsperipheral_target_type - the sensor or effector structure/organ targeted by an afferent/efferent neuron.body_part_sensory - the part of the body innervated by an afferent neuronbody_part_effector - the part of the body targeted by an efferent neuron. If known, this is the site of action when it is different from the body part innervated (e.g. wing power motor neurons innervate muscles located in the thorax but move the wing)nerve - peripheral nerve (if applicable)hemilineage - developmental lineage (NA for many neurons)neurotransmitter_verified/neuropeptide_verified - neurotransmitter/neuropeptide of neuron, as reported in the literaturefafb_783_match_id/manc_121_match_id - segment ID of neuron from FAFB v783/MANC v1.2.1 that matches the BANC neuronneurotransmitter_predicted - CNN-predicted primary neurotransmitterother_names - names given to the neuron that are not the cell_type name
Supplementary Data 2: Updated annotations for FAFB Brain Neurons
Contains metadata for brain neurons from the FAFB-FlyWire dataset that are integrated into BANC analyses. This enables comparison and integration between the BANC neck connective data and the comprehensive adult brain connectome. Cell type names are unchanged.
root_783 - FlyWire neuron ID (root_id in FAFB dataset release 783)nerve - peripheral nerve (if applicable)hemilineage - developmental lineage (NA for many neurons)region - region of the CNS; all neurons that have arbors in the optic lobe are considered optic_lobe and all neurons that fully transit the neck connective between the brain and VNC are considered neck_connectiveflow - from the perspective of the whole CNS, whether the neuron is afferent, efferent or intrinsicsuper_class - coarse division, hierarchical below flowcell_class - hierarchical below super_classcell_sub_class - hierarchical below cell_classcell_type - Individual cell type name (e.g., ORN_DM6, ORN_VA1v). Not modified from original projectneurotransmitter_predicted - CNN-predicted primary neurotransmitter^46^neurotransmitter_verified - neurotransmitter, as reported in the literature
Supplementary Data 3: Updated annotations for MANC VNC Neurons
Contains metadata for ventral nerve cord neurons from the MANC dataset that are integrated into BANC analyses. This enables comparison and integration between the BANC neck connective data and the comprehensive adult VNC connectomes. Cell type names unchanged.
bodyid - MANC neuron ID from v1.2.1nerve - Peripheral nerve association (if applicable)hemilineage - Developmental lineage (NA for many neurons)region - region of the CNS; all neurons that have arbors in the optic lobe are considered optic_lobe and all neurons that fully transit the neck connective between the brain and VNC are considered neck_connectiveflow - from the perspective of the whole CNS, whether the neuron is afferent, efferent or intrinsicsuper_class - coarse division, hierarchical below flowcell_class - hierarchical below super_classcell_sub_class - hierarchical below cell_classcell_type - Individual cell type name (e.g., SNpp50, IN19A001). Not modified from original projectneurotransmitter_predicted - CNN-predicted primary neurotransmitterneurotransmitter_verified - neurotransmitter, as reported in the literature
Supplementary Data 4: ANs and DNs with UMAP coordinates and cluster assignments
Contains the ANs and DNs, along with their functional clustering based on connectivity patterns (Fig. 3a)
root_id - BANC neuron identifier when used in analysisroot_626 - BANC release v626 specific identifiersupervoxel_id - supervoxel identifier for positionposition - 3D coordinates in BANC space (x, y, z in BANC raw voxel space)UMAP1, UMAP2 – 2D embedding coordinates from connectivity-based UMAP analysisside - from the fly’s perspective, the side on which the cell body is locatedregion - region of the CNS (primarily neck_connective)nerve - peripheral nerve (if applicable)super_class - ascending, descending. Note, we only included flow == ‘intrinsic’ neuronshemilineage - developmental lineagecell_function - functional role description from our literature reviewcluster - cluster assignment from Fig. 3a. The number defines the cluster identity. Note that ANs have AN_ appended in front of the number and DNs have DN_ appended, but cells with the same number belong to the same cluster, regardless of the prefixsuper_cluster - AN/DN superclusters, the named cluster amalgamations used in this paper’s figurescell_type - BANC-specific cell type name, for DNs this preferentially comes from FAFB, for ANs from MANCfafb_cell_type - corresponding cell type in FAFB datasetmanc_cell_type - corresponding cell type in MANC dataset
Supplementary Data 5: Effector cells with UMAP coordinates and functional cluster assignments
Contains all efferent neurons, clustered by their functional properties and target effector systems (Fig. 2i). These neurons control movement, secretion and other output functions.
root_id - BANC neuron identifier when used in analysisroot_626 - BANC release v626 specific identifiersupervoxel_id - supervoxel identifier for positionposition - 3D coordinates in BANC space (x, y, z in BANC raw voxel space)UMAP1, UMAP2 – 2D embedding coordinates from connectivity-based UMAP analysisside - from the fly’s perspective, the side on which the cell body is locatedregion - region of the CNSnerve - peripheral nervesuper_class - efferent type (motor, visceral_circulatory)hemilineage - developmental lineagecell_function - functional role (e.g. leg_motor, antenna_motor, neck_motor).cluster - cluster assignment from Fig. 2i, as the cluster number with EFF_ appended (e.g., EFF_01)super_cluster - effector cell groups, the named cluster amalgamations used in this paper’s figures.cell_type - BANC-specific cell type namefafb_cell_type - corresponding cell type in FAFB datasetmanc_cell_type - corresponding cell type in MANC dataset
Supplementary Data 6: CNS network analysis with spectral clustering and UMAP embedding
Contains neurons from spectral clustering analysis of the CNS connectivity (Fig. 6a), revealing network-level organisation beyond individual cell types. This analysis identifies functional networks that span multiple brain regions.
root_id - BANC neuron identifier when used in analysisroot_626 - BANC release v626 specific identifiersupervoxel_id - supervoxel identifier for positionposition - 3D coordinates in BANC space (x, y, z in BANC raw voxel space)UMAP1, UMAP2 – 2D embedding coordinates from connectivity-based UMAP analysisside - from the fly’s perspective, the side on which the cell body is locatedregion - region of the CNSnerve - peripheral nerve (if applicable)super_class - high-level functional category (various types including visual_projection, central_brain_intrinsic)hemilineage - developmental lineagecell_function - functional description (if known)cluster - effector clusters (from Fig. 2i), which have the EFF_ prefix, and AN/DN clusters (from Fig. 3a), which have the AN_ or DN_ prefix (if applicable)super_cluster - name of effector cell group or AN/DN supercluster (if applicable)cns_network - CNS networks as determined by spectral clustering, 13 cluster cutcell_type - BANC-specific cell type namefafb_cell_type - corresponding cell type in FAFB datasetmanc_cell_type - corresponding cell type in MANC dataset
Supplementary Data 7: Literature review on cell function for ascending, descending and visual projection neurons
Cell_type - cell type names in the BANC connectomeOther_names - other names used for this cell type in the literaturesuper_class - high-level functional category, here only ascending, descending and visual projectionCell_function - simple descriptive label for the ‘function’ of the cell typeCitations - short hand citations for the work that helped determine cell_function

## Data Availability

Data is freely accessible through multiple platforms. A general overview of the resource and links to these tools are available at the BANC portal (https://banc.community). The FlyWire Codex^280^ (https://codex.flywire.ai/banc) provides an interactive web interface for exploring the BANC connectome, enabling users to search for neurons, visualize morphology, traverse synaptic pathways and download metadata such as cell-type annotations, neurotransmitter predictions and connectivity matrices. Volumetric EM data, including 3D neuron meshes and annotations, can be viewed at https://ng.banc.community/view or accessed programmatically via CAVE^33^. We snapshotted CAVE materialization version 626 (July 21, 2025) for this manuscript. Static data dumps are also available for download from the Harvard Dataverse (https://doi.org/10.7910/DVN/8TFGGB). Direct downloads include: the synaptic connectivity edgelist, NBLAST results of BANC neurons against Hemibrain, FAFB, FANC and MANC as well as BANC all-by-all; neuronal L2 skeletons (made using: https://github.com/CAVEconnectome/pcg_skel); neuronal colorMIPs; influence scores from defined sources as used in this manuscript and our aligned BANC metadata. Schematics are available here as vector graphics: https://github.com/wilson-lab/schematics?tab=readme-ov-file.

## Code availability

All code developed for this project is open-source and publicly available. Our connectome data is most accessible through FlyWire codex, where it is browsable and from where up-to-date direct downloads can be obtained, as the project progresses (https://codex.flywire.ai/?dataset=banc). A comprehensive collection of community tools and software packages for working with the BANC dataset can be found at the project hub (https://banc.community) and the FlyWire Apps portal (https://flywire.ai/apps). The specific code used to perform the analyses and generate the figures for this manuscript is shared in a dedicated GitHub repository: https://github.com/htem/BANC-project/. Code for computing influence scores is available at: https://doi.org/10.5281/zenodo.15999930^271^. Code for neurotransmitter predictions is available at: https://github.com/htem/synister_banc. We have also made available python code for BANC (https://pypi.org/project/banc/), and an R package, bancr (https://github.com/flyconnectome/bancr), for querying BANC data, compatible with the natverse^78^. A static snapshot of the code and analysis tools are also available on our Harvard Dataverse Dataset (https://doi.org/10.7910/DVN/8TFGGB).
